# Integrative comparative genomics and transcriptomics reveal key roles of *SAG17* and *SAG23* in early-stage virulence divergence of *Eimeria tenella*

**DOI:** 10.1186/s13567-026-01730-0

**Published:** 2026-04-28

**Authors:** Ye He, Xiang Wan, Xuan Wang, Ying Chen, Dan He, Yongmei Yu, Shunping Dong, Mingjie Wu, Liting Cao, Bi Wang

**Affiliations:** 1 College of Animal Science, Guizhou University, Guiyang, 550025 Guizhou People’s Republic of China; 2 Chongqing Three Gorges Vocational College, Wanzhou, Chongqing, 404155 People’s Republic of China; 3 Key Laboratory of Animal Diseases and Veterinary Public Health of Guizhou Province (Cultivation), Guiyang, 550025 Guizhou People’s Republic of China; 4 Department of Traditional Chinese Veterinary Medicine, College of Veterinary Medicine, Southwest University, Chongqing, 402460 People’s Republic of China; 5 Institute of Animal Husbandry and Veterinary Medicine of Guizhou Academy of Agricultural Sciences, Guiyang, 550025 Guizhou People’s Republic of China; 6 College of Forestry, Guizhou University, Guiyang, 550025 Guizhou People’s Republic of China; 7 Key Laboratory of Animal Genetics, Breeding and Reproduction in the Plateau Mountainous Region, Ministry of Education, Guizhou University, Guiyang, 550025 Guizhou People’s Republic of China

**Keywords:** *Eimeria tenella*, virulence divergence of geographic strains, resequencing, transcriptome analysis, in vitro intervention experiment

## Abstract

*Eimeria tenella* is one of the major pathogens threatening the poultry industry. However, the molecular mechanisms underlying the divergence of virulence among geographic strains remain poorly understood. In this study, clinical phenotypes, histopathology, cytokine assays, whole-genome resequencing, and transcriptome analyses were integrated to systematically compare the Beijing and Guizhou strains. The results showed that infection with the Guizhou strain caused more severe clinical symptoms and intestinal lesions in chickens, accompanied by stronger interleukin (IL)-17 and tumor necrosis factor (TNF)-α responses. Genomic variation analysis revealed abundant nonsynonymous and promoter mutations in the surface antigen (SAG) membrane protein family of the Guizhou strain, and transcriptomic data further confirmed high sporozoite-stage expression of *SAG5*, *SAG13*, *SAG17*, and *SAG23*, which strongly correlated with virulence phenotypes. Functional validation demonstrated that the recombinant SAG17 and SAG23 proteins significantly inhibited invasion by the *E. tenella* sporozoites and enhanced levels of the host’s interferon (IFN)-γ and immunoglobulin G (IgG), with SAG17 showed stronger immunostimulatory effects than SAG23. Collectively, this study identified SAG17 and SAG23 as key molecules driving virulence divergence of *E. tenella* geographic strains, providing molecular evidence for the mechanisms of parasite virulence and highlighting important targets for the development of novel vaccines and anticoccidial drugs.

**Graphical Abstract:**

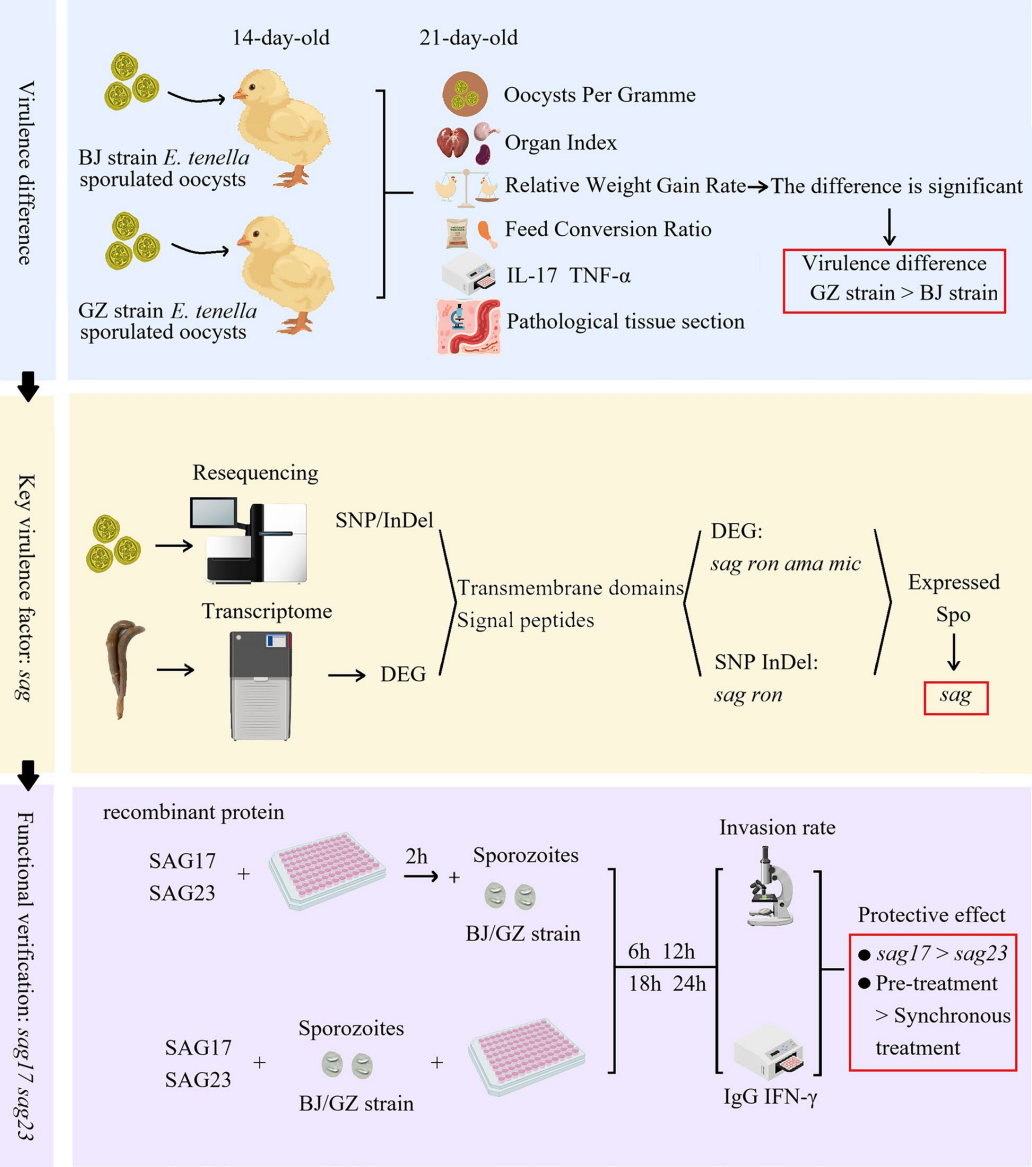

**Supplementary Information:**

The online version contains supplementary material available at 10.1186/s13567-026-01730-0.

## Introduction

Coccidiosis in chickens is an important infectious disease caused by protozoan parasites of the genus *Eimeria* [1]. This disease has been estimated to cause more than 13.5 billion US dollars in economic losses annually to the global poultry industry [2], making it one of the most devastating diseases in poultry production. Among *Eimeria* species, *Eimeria tenella* is the most pathogenic to chickens [3]. It specifically parasitizes the cecum [4], leading to thickening of the cecal wall, shortening, rupture, or even complete loss of villi, accompanied by severe diarrhea, bloody stools, depression, and anorexia [5, 6]. These pathological changes markedly impair nutrient absorption, resulting in weight loss and, in severe cases, death of the host [7].

The process of *Eimeria* infection in chickens is the result of precise molecular interactions between the parasite and host cells and mainly involves four stages: (i) initiation of infection, triggered by excystation of sporulated oocysts and the release of sporozoites; (ii) invasion of sporozoites into intestinal epithelial cells of the host; (iii) intracellular development regulated at the molecular level, including schizogony and gametogony; and (iv) development of *Eimeria* oocysts into mature oocysts within host cells followed by the release of oocyst-shedding factors, thereby triggering oocyst detachment from host cells and subsequent excretion from the host. Previous studies have indicated that the invasive capacity of sporozoites largely determines the subsequent proliferation efficiency of coccidia and the extent of host tissue damage [8, 9]. Membrane proteins are key mediators of host–parasite interactions and serve as major targets for vaccine development owing to their abundant surface expression and strong immunogenicity [10]. This group of proteins includes rhoptry neck proteins (RONs), apical membrane antigens (AMAs), microneme proteins (MICs), and surface antigens (SAGs). As the earliest parasite-derived molecules encountered by the host immune system during *E. tenella* infection, these membrane proteins function in host-cell recognition and invasion, and also contribute to immune evasion and the regulation of parasite virulence [11–14].

SAGs are membrane-bound proteins present on the surface of invasive sporozoites and merozoites, anchored to the phospholipid bilayer through glycosylphosphatidylinositol (GPI) [15]. Their conserved surface domains, such as cysteine-rich regions, bind nonspecifically to sulfated proteoglycans on host-cell surfaces, thereby mediating the initial adhesion of *E. tenella* [16]. Following adhesion, SAGs undergoes conformational changes and act in concert with virulence factors such as RONs and MICs to induce host-cell membrane invagination [17] and trigger signaling pathways, including calcium and mitogen-activated protein kinase (MAPK) cascades, which drive active parasite invasion while shortening the developmental cycle, ultimately promoting parasite survival [18, 19]. In addition, SAGs coordinate host immunity by activating nuclear factor kappa-light-chain-enhancer of activated B cells (NF-κB)-dependent cytokine responses (IL-17, IL-12, and IFN-γ), while simultaneously suppressing Janus kinase/signal transducer and activator of transcription (JAK–STAT) signaling to facilitate immune evasion [20–22].

Previous studies have demonstrated marked regional differences in the pathogenicity of *Eimeria* species [23]. Consistently, our clinical observations further revealed that chickens infected with the Beijing and Guizhou strains of *E. tenella* exhibited significant variation in clinical manifestations, including the severity of bloody diarrhea, lethargy, and reduced feed intake. On the basis of previous studies demonstrating that membrane proteins play critical roles in determining host damage during parasite invasion and colonization, it was hypothesized that the clinical differences observed between strains might can be attributed to variations in membrane proteins. The objectives of this study are: (i) to determine the differential effects of the Beijing and Guizhou strains on host pathology; and (ii) to identify the molecular mechanisms underlying these differences, with a focus on the potential roles of membrane proteins. This study integrated whole-genome resequencing and transcriptomic analyses to compare the Beijing and Guizhou strains of *E. tenella*, uncovering the molecular mechanisms underlying their virulence divergence and providing a foundation for precision vaccine design and targeted anticoccidial strategies.

## Materials and methods

### Ethics statement

All experiments involving animals were approved by the Experimental Animal Welfare Ethics Review Committee of Guizhou University (EAE-GZU-2024-E058, EAE-GZU-2024-T069).

### Parasite strains

Purified oocysts of the Guizhou strain of *E. tenella* were obtained from the stock maintained at the College of Animal Science, Guizhou University. Purified oocysts of the Beijing strain of *E. tenella* were kindly provided by Professor Xianyong Liu from the Department of Parasitology, China Agricultural University.

### Animal husbandry

One-day-old male *cyan-shank partridge* chickens were purchased from Guizhou Shanshui Qiannong Breeding Co., Ltd (Guizhou, China). The chickens were reared in dedicated isolators under controlled environmental conditions (temperature 33–35 °C, humidity 60–65%, light/dark cycle of 16 h/8 h). Sterilized feed and sterile water were provided ad libitum throughout the experimental period. The poultry house was disinfected twice daily for seven consecutive days before chicken introduction. The behavior, feed intake, and health status of the chickens were monitored daily, and an animal welfare assessment system was implemented.

### Sample collection

#### Whole-genome resequencing samples

Eighty 15-day-old chickens were randomly allocated into two groups (*n* = 40 each) and orally infected with either the Beijing or Guizhou strain of *E. tenella* at a dose of 1 × 10^5^ sporulated oocysts per chicken. At 7 days post-infection (dpi), cecal tissues were scraped, and oocysts were purified and recovered. Purified oocyst pellets of the Beijing and Guizhou strains were collected, with three tubes (0.5 mL each) prepared for each strain, then stored at −80 °C for subsequent whole-genome resequencing analysis.

#### Transcriptome samples

A total of 135 15-day-old chickens were used. Fifteen chickens orally received an equal volume of phosphate-buffered saline (PBS) and were designated as the control group (CON). Sixty chickens were orally infected with the Beijing strain of *E. tenella* at a dose of 1.5 × 10^4^ sporulated oocysts per chicken, designated as the Beijing strain infection group (BSIG). Another 60 chickens were infected under the same conditions with the Guizhou strain and designated as the Guizhou strain infection group (GSIG).

On the basis of microscopic examination of cecal tissue sections, four key developmental stages of *E. tenella* were accurately identified: the sporozoite stage (16 days old, Spo), the schizogony stage (19 days old, Sch), the gametogony stage (21 days old, Gam), and the peak of oocyst shedding (22 days old, Pos). At each stage, 15 cecal samples were collected from different individuals on one side of the cecum (the contralateral cecum was reserved for lesion scoring and pro-inflammatory cytokine detection). The cecum was longitudinally opened, and luminal contents were gently removed using RNase-free PBS. All samples were stored at −80 °C for transcriptome sequencing analysis.

The 15 samples collected at each time point were pooled into three subgroups, each consisting of mixed samples from five individuals. This approach minimized stochastic variation and enhanced the representativeness of infection-induced transcriptional changes at the population level. On the other hand, a pooled-sample strategy was adopted to achieve a reasonable balance between sequencing cost and depth, thereby improving the detection efficiency of low-abundance genes. This approach has been widely applied in studies focusing on population-level differences rather than individual variation [4]. Although this approach was unable to capture individual-specific expression profiles or quantify interindividual expression variability, it was selected because the present study was focused on transcriptional differences at the population level induced by *E. tenella* infection.

### Comparison of clinical parameters

The BSIG and GSIG groups were each composed of three independent cages, with 20 chickens housed per cage. The clinical phenotypes and behavioral conditions were continuously monitored to ensure the reproducibility and reliability of the observations. The phenotypic data included body weight, average feed intake, average body weight gain, relative weight gain rate (RWGR), feed conversion ratio (FCR), oocyst output (oocysts per gram, OPG), and organ indices (liver indices, LI; spleen indices, SI; and bursa of Fabricius indices, BOFI). The measurement methods for each parameter were as follows:

(1) Body weight gain rate was assessed by randomly selecting five chicks per cage, which were weighed before infection and at defined post-infection time points to monitor dynamic changes in individual body weight.

Relative weight gain rate (RWGR) = (weight gain of each experimental group/weight gain of the negative control group) × 100%.

(2) Feed intake was recorded for each replicate cage as total feed consumption and subsequently normalized to per-chicken intake on the basis of 20 chickens per cage, thereby minimizing the influence of stocking density on the data.

Feed conversion ratio (FCR) = average feed intake/average body weight gain.

(3) Oocyst output (OPG) counts were determined using a five-point sampling method. Fecal samples were collected from five predefined regions (upper, lower, left, right, and center) of the fecal tray in each cage. The samples were thoroughly mixed prior to oocyst counting to ensure representativeness.

Oocyst output (OPG) = number of oocysts per gram of feces, determined by the McMaster counting method [24].

(4) After necropsy, organ weights (including the liver, spleen, and bursa of Fabricius) were measured, and organ indices were calculated accordingly to assess organ changes.

Organ index (OI) = organ weight (liver, spleen, or bursa of Fabricius)/body weight.

### Histomorphological examination

Cecal tissues from chickens in different treatment groups were examined using the paraffin section method [25]. The microscope used was an Olympus BX53 (Olympus Corporation, Tokyo, Japan).

### Cytokine detection

At the four developmental stages of the Beijing and Guizhou strains (Spo, Sch, Gam, and Pos), as well as in the CON group, cecal tissues from three randomly selected chickens were collected from one side of the cecum. The cecal tissues were excised, gently rinsed with physiological saline, placed into centrifuge tubes, and homogenized with grinding beads in an appropriate volume of saline. The homogenates were centrifuged at 3500 rpm for 15 min, and the resulting supernatant was collected. Levels of interleukin-17 (IL-17) and tumor necrosis factor-α (TNF-α) were measured using an enzyme-linked immunosorbent assay (ELISA). The absorbance (optical density (OD) value) was detected at a wavelength of 450 nm. The ELISA kits (kit nos. MM-054801, MM-093801) for chicken IL-17 and TNF-α were purchased from Jiangsu Meimian Industrial Co., Ltd. (Jiangsu, China). Each experiment was independently repeated three times, with three technical replicates per biological replicate.

### Whole-genome resequencing analysis

Genomic DNA was extracted using the magnetic bead method (QIAamp DNA Mini Kit, Qiagen). The concentration, quality, and integrity of the DNA were assessed using a NanoDrop 2000 spectrophotometer. Following fragmentation, the desired DNA fragments were recovered by electrophoresis and sequencing libraries were created through adaptor ligation. Sequencing was then performed on the Illumina NovaSeq 6000 platform.

Clean reads were aligned to the *E. tenella* Houghton strain (selected as reference genome owing to its high level of annotation completeness; although it exhibits genetic divergence from the local strain, this does not compromise the reliability of variant detection; GenBank accession no. GCF_000499545.2) [26] using Bowtie2 (version 2.3.5) [27], and the alignment results were sorted using SAMtools (version 1.15) [28]. Single-nucleotide polymorphism (SNP) calling was performed using SAMtools mpileup and VarScan (version 2.4.3) [29] with the following parameters: -min-coverage 20, -min-reads2 5, and -min-freq-for-hom 0.75. Variant annotation was conducted using SnpEff (version 4) [30] to identify gene associations and synonymous and nonsynonymous mutations and their potential impact on amino acid sequences. Nonsynonymous (missense) mutations, promoter-region variants, and InDel loci were filtered and retained. These loci were then used to construct a functional gene set at the DNA level for subsequent analysis. The raw resequencing data were deposited in the National Center for Biotechnology Information (NCBI) database under accession nos. SRR35396008 for the Beijing strain and SRR35396007 for the Guizhou strain.

### Transcriptome analysis

Total RNA was extracted using the TRIzol reagent (Invitrogen Life Technologies), and RNA purity, concentration, and integrity (RIN ≥ 7) were assessed with a NanoDrop 2000 spectrophotometer. Eukaryotic mRNA with poly(A) tails was enriched and purified from total RNA using oligo(dT) magnetic beads. The enriched mRNA was randomly fragmented and reverse-transcribed to synthesize complementary DNA (cDNA), followed by adaptor ligation to construct sequencing libraries. These libraries were sequenced on an Illumina NovaSeq 6000 platform.

Raw reads were filtered using Trimmomatic (version 0.36) [31] for data processing to obtain high-quality clean reads. The filtered sequences were then aligned to the *E. tenella* ETH001 reference genome (GenBank accession no. GCF_000499545.2) [26] using HISAT2 (version 2.2.1) [32]. Gene expression levels were quantified as fragments per kilobase of transcript per million mapped reads (FPKM) [33]. The raw count matrix was generated from the alignment files using HTSeq (version 0.11.2) [34], which served as the input for differential expression analysis. Differentially expressed genes (DEGs) were identified using edgeR (version 3.26.6) [35] after trimmed mean of M values (TMM) normalization. Genes with |Log_2_ (fold change)|> 1 and a false discovery rate (FDR) < 0.05 were considered to be significantly differentially expressed.

All membrane protein sequences were identified on the basis of the annotated coding sequences of the reference genome. All of the predicted protein-coding sequences of the differentially expressed genes (DEGs) were analyzed using SignalP 5.0 [36] to identify signal peptides. Those containing signal peptides were then analyzed using TMHMM 2.0 [37] to identify membrane-associated motifs. Genes encoding proteins with both predicted N- (signal peptide) and C-terminal (GPI anchor to membrane) regions were classified as membrane protein-related genes. The raw transcriptome data were deposited in the National Center for Biotechnology Information (NCBI) database under accession nos. PRJNA1163523 for the Beijing strain and PRJNA1327252 for the Guizhou strain. Pearson correlation analysis was performed to integrate the transcriptome and resequencing data, thereby elucidating the spatiotemporal expression dynamics of *E. tenella* membrane protein-related genes.

### Quantitative validation of key membrane protein genes

Four key membrane protein genes were validated by quantitative PCR (qPCR) (primer sequences listed in Table 1). RNA extraction was performed as described in “Transcriptome analysis” section. and cDNA synthesis was carried out using a reverse transcription kit (TaKaRa, code no. RR047A, Japan) according to the manufacturer’s instructions. A mixture of oligo dT and random hexamer primers was used for reverse transcription.

**Table 1:** **Primer sequences used for qPCR**

Primer name	Sequences (5′ → 3′)
18s-F	TGTAGTGGAGTCTTGGTGATTC
18s-R	CCTGCTGCCTTCCTTAGATG
*sag5*-F	TATGCGTTCAAGTCCCTCAC
*sag5*-R	TTGTTGCTCCGACCCTCC
*sag13*-F	CTTCCCTCGCACAAGTAGCA
*sag13*-R	CAGCAACCCATTCAGTCCCT
*sag17*-F	AGGGACCGATGCGGATAA
*sag17*-R	ATGGGTCAGTCCTGTGGTAAAG
*sag23*-F	TGTTACGCTGGTCTTCTCG
*sag23*-R	GCCAGGCAATCATTAGTTTT

The qPCR reactions were performed in a total volume of 20 μL, containing 10 μL of Universal Blue SYBR Green qPCR Master Mix (Biosharp, Canada), 0.8 μL of forward primer (0.25 μM), 0.8 μL of reverse primer (0.25 μM), 1 μL of cDNA template (100 ng/μL), and 7.4 μL of diethyl pyrocarbonate (DEPC)-treated water. The thermal cycling conditions were as follows: 95 °C for 30 s (initial denaturation), followed by 35 cycles of 95 °C for 15 s (denaturation) and 60 °C for 30 s (annealing/extension). qRT-PCR was performed on an FQD-96A Real-Time PCR system (Bioer, Hangzhou, China). Primers were designed using Primer5 software, and their specificity was assessed with the Primer3 tool available at NCBI. The housekeeping gene 18S rRNA was used as the internal reference. Relative gene expression levels were calculated using the 2^−ΔΔCT^ method [38].

### Construction of prokaryotic expression vectors for *SAG17*/*SAG23* and protein purification

On the basis of the high expression during the sporozoite stage and strong correlation with clinical phenotypes, *SAG17* and *SAG23* were selected for recombinant protein expression. The gene sequences of *E. tenella SAG17* (GenBank accession no. AJ586541.1) and *SAG23* (GenBank accession no. AJ586547.1) were retrieved from NCBI, codon-optimized, and synthesized. Regarding adoption of the targeted expression, only the central PDB functional domain (the predicted core region) was cloned and expressed, with an N-terminal 6× His tag introduced to enable efficient purification. This design was intended to retain the key functional/antigenic region while improving recombinant expression performance and solubility. The coding sequences (CDSs) of *SAG17* and *SAG23* used for functional validation were identical to those of the reference strain. The optimized sequences were cloned into the pCZN1 vector (for detailed information, see [39]; Zoonbio Biotechnology Co., Ltd., Jinan, China) to construct recombinant plasmids pCZN1-*SAG17* and pCZN1-*SAG23*. The plasmids were transformed into *Escherichia coli* TOP10 competent cells, extracted, and verified by double digestion with *Nde*I and *Xba*I.

Verified recombinant plasmids were subsequently transformed into *E. coli* Arctic Express cells. The transformed strain was cultured at 37 °C with shaking until the OD_600_ reached 0.6–0.8, after which protein expression was induced with 0.2 mM isopropyl β-d-1-thiogalactopyranoside (IPTG) for 4 h. Bacterial pellets were harvested and lysed by sonication, and both supernatant and precipitate fractions were analyzed by 12% sodium dodecyl sulfate (SDS)-polyacrylamide gel electrophoresis (PAGE) (Vazyme, MP102-02). Tagged protein was purified by Ni-nitrilotriacetic acid (NTA) affinity chromatography. The purified products were further analyzed by reducing SDS-PAGE and transferred onto polyvinylidene fluoride (PVDF) membranes, followed by blocking with 5% skimmed milk, incubation with primary antibody (anti-N-terminal 6× His tag monoclonal antibody, Zoonbio Biotechnology Co., Ltd., Jinan, China.) and secondary antibody (horseradish peroxidase (HRP)-conjugated goat anti-mouse IgG), and visualization using enhanced chemiluminescence (ECL) reagent (WB, Thermo Fisher, 26620). Protein identity and molecular weight were confirmed by liquid chromatography–mass spectrometry (LC–MS), and the purified proteins were stored at −80 °C. The recombinant proteins were initially obtained in the form of inclusion bodies. To facilitate protein refolding, a stepwise refolding procedure was applied. Inclusion bodies were first solubilized using urea in the presence of dithiothreitol (DTT) to disrupt mispaired disulfide bonds and protein aggregates. The denaturant was then gradually removed by stepwise dialysis to provide a mild refolding environment. Finally, the proteins were dialyzed into PBS buffer (pH 7.4), yielding soluble recombinant proteins without residual denaturants that could interfere with subsequent experiments. Negative controls were included throughout the procedure to ensure specificity [40, 41].

### Isolation, culture, and identification of primary cecal epithelial cells (PCECs)

Cecal tissues were aseptically collected from 18-day-old chicken embryos, rinsed with PBS, and mechanically minced into ~1-mm^3^ fragments. The tissues were digested in PBS containing 1 mg/mL collagenase I at 37 °C for 70 min. The reaction was terminated, and the suspension was centrifuged stepwise to remove the digestive solution. The samples were subjected to three rounds of PBS suspension–static precipitation cycles, and the supernatants were collected. The cells were then pelleted by centrifugation at 1600 rpm for 3 min, washed three times, and resuspended to obtain the purified cell pellets. The cell density was adjusted to 1 × 10^5^ cells/mL in epithelial cell complete medium supplemented with 10% fetal bovine serum (FBS). The cells were seeded into T25 flasks for routine culture at 37 °C in a humidified incubator with 5% CO_2_. Cell adhesion efficiency (>85%) was confirmed by microscopy after 24 h.

For immunofluorescence identification, three coverslips were placed in a 24-well plate, each well with 1 mL of medium and 0.02 × 10^6^ cells. The plate was then incubated for 2 h. After cell adherence, the medium was aspirated, and the cells were washed once with PBS, fixed in 4% paraformaldehyde at 4 °C for 30 min, and rinsed three times with PBS (5 min each). Coverslips were air-dried and placed cell-side up on a support within culture dishes. Fifty microliters of permeabilization/blocking buffer were added on a hydrophobic membrane, and coverslips were inverted onto the droplets for 2 h. Subsequently, 50 μL of the primary antibody (1:100–1:200 dilution in PBS) was applied, and coverslips were incubated at 4 °C overnight. After washing, the cells were incubated with the secondary antibody (1:500 dilution in PBS) at room temperature for 2 h in the dark, followed by three washes with PBS (5 min each). The nuclei were counterstained with 4′,6-diamidino-2-phenylindole (DAPI; 1:1000 dilution in PBS) for 5 min and washed three times with PBS (5 min each time). Finally, the coverslips were mounted with one drop of Fluoromount-G on the cell-bearing surface [42, 43].

### Extraction of *E. tenella* sporozoites

The sporulated oocysts were surface-sterilized with 30% sodium hypochlorite for 20 min, after which they were mechanically disrupted by vortexing with 1-mm glass beads for 2–3 min. After removing the glass beads, the oocyst suspension was incubated at 42 °C in digestion buffer for 40–60 min. To prepare the digestion buffer, 1 mL of fresh chicken bile was mixed with 0.075 g of trypsin and brought to a final volume of 10 mL using sterile PBS. The solution was then homogenized, filtered through a 0.22-μm membrane, aliquoted, and stored at −20 °C. The reaction was terminated once the rate of sporozoite excystation reached ≥ 80%, as confirmed by microscopy. The sporozoites were then purified using a sterile G3 funnel filter, adjusted to a concentration of 1 × 10^5^ sporozoites/mL, and stored at 4 °C until required [44].

### In vitro protein intervention assay

Primary cecal epithelial cells (PCECs) were seeded into 96-well plates and subsequently subjected to intervention assays (Table 2). A blank control (BC) group containing only culture medium was also set up. Additionally, two control groups were set up: a Beijing strain control group (BSCG), inoculated with 1 mL of *E. tenella* Beijing strain sporozoites (1 × 10^5^ sporozoites/mL), and a Guizhou strain control group (GSCG), inoculated with 1 mL of *E. tenella* Guizhou strain sporozoites (1 × 10^5^ sporozoites/mL).

**Table 2:** **Experimental groups for the in vitro sporozoite invasion assay with recombinant protein intervention**

Source of parasite strain	Group name	Treatment method
Beijing strain *E. tenella*	BSCG	–
	BP-17	SAG17 pretreatment PCEC
	BP-23	SAG23 pretreatment PCEC
	BS-17	SAG17-synchronized PCEC
	BS-23	SAG23-synchronized PCEC
Guizhou strain *E. tenella*	GSCG	–
	GP-17	SAG17 pretreatment PCEC
	GP-23	SAG23 pretreatment PCEC
	GS-17	SAG17-synchronized PCEC
	GS-23	SAG23-synchronized PCEC

*Eimeria tenella* (*E. tenella*)

Cells were first incubated with 5 µg of recombinant protein for 2 h, followed by inoculation with 1 mL of *E. tenella* sporozoites from either the Beijing or Guizhou strain to establish the pretreatment groups, which were designed to simulate a preventive scenario prior to infection. Recombinant protein (5 μg) and 1 mL of sporozoites from the Beijing or Guizhou strain were added to the cells simultaneously to model an emergency post-infection intervention. The detailed grouping information is provided in Additional file 2.

All groups were cultured at 37 °C with 5% CO_2_ for 6, 12, 18, and 24 h. At each time point, culture plates were centrifuged at 1500 rpm for 5 min, the supernatants were collected, and the cell pellets were resuspended in PBS for parasite counting. A 10-μL suspension of sporozoites was aspirated using a sterile pipette and then slowly added into a McMaster counting chamber. The preparation was allowed to stand for 3–5 min before counting. At 24 h, supernatants were further analyzed using chicken interferon-γ (IFN-γ) and immunoglobulin G (IgG) ELISA kits (Jiangsu Meimian Industrial Co., Ltd., China) to quantify IFN-γ (kit no. MM-050501) and IgG (kit no. MM-052001) levels. These measurements were used to evaluate the impact of the recombinant SAG17 and SAG23 proteins on the invasion of cecal epithelial cells by *E. tenella* sporozoites.

Each experiment was independently repeated three times, with three technical replicates per biological replicate, to ensure the robustness and reproducibility of the results.

### Data processing and visualization

The data were organized and preliminarily analyzed using Microsoft Excel. Statistical analyses were performed using SPSS 20.0 software. Analysis of variance (ANOVA) was conducted, followed by a Duncan’s multiple range test for pairwise comparisons. Differences were considered statistically significant at *p* < 0.05. Data visualization was carried out using GraphPad Prism 10 (San Diego, CA, USA), Hiplot (Shanghai Tengyun Biotechnology Co., Ltd., Shanghai, China), and MicroBioInfo (Shanghai Newcore Biotechnology Co., Ltd., Shanghai, China). Results are expressed as mean ± standard deviation (SD).

## Results

### Clinical phenotypes

**Figure 1:**
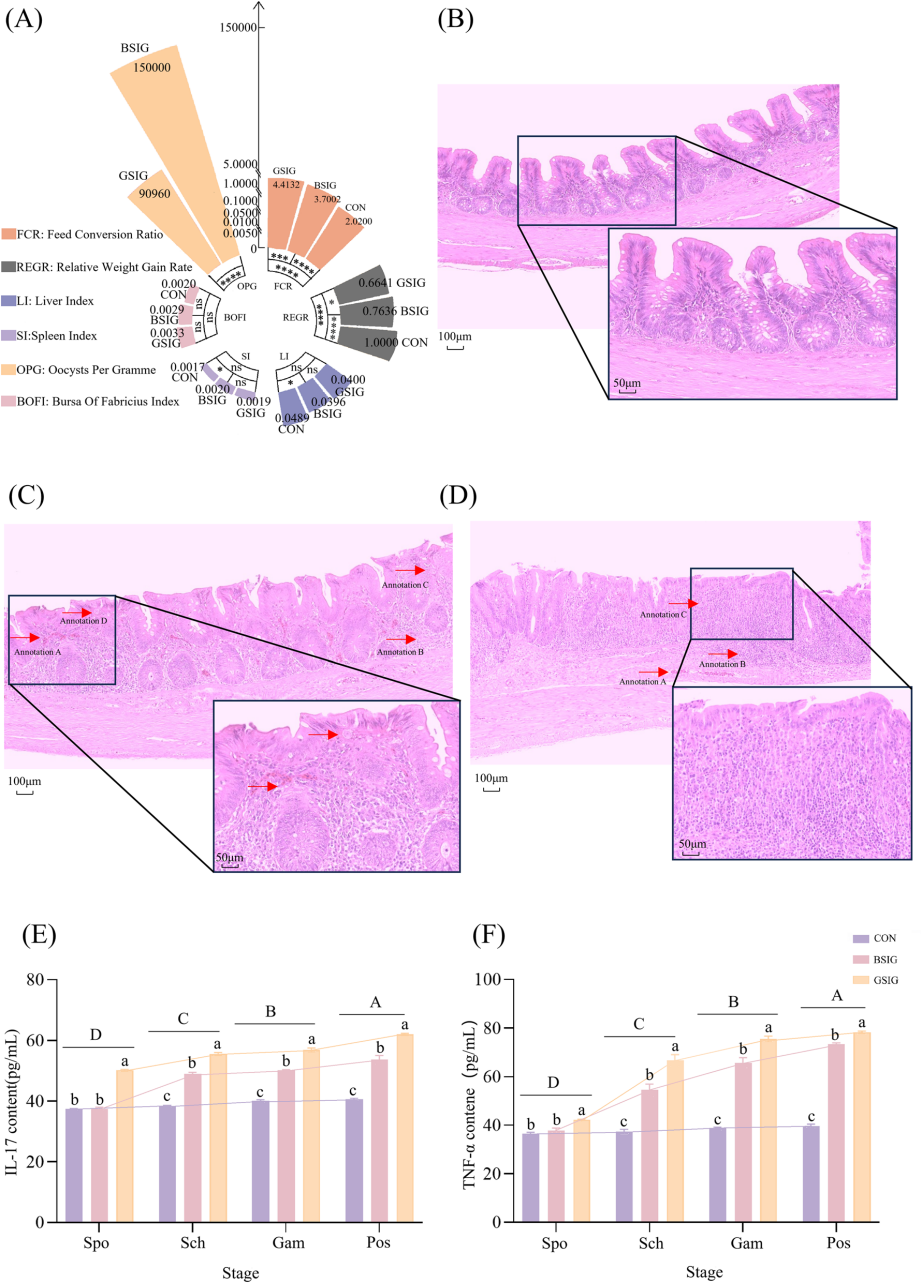
**Clinical phenotypes and inflammatory responses of chickens infected with *Eimeria tenella.*** **A** The clinical manifestation results. **B**–**D** Histological sections of cecal tissues from CON, BSIG, and GSIG groups at the *E. tenella* peak of oocyst shedding stage. **E**, **F** Levels of IL-17 and TNF-α in cecal tissues. The *y*-axis represents the concentrations of IL-17 and TNF-α (pg/mL), and the *x*-axis denotes the four developmental stages of infection. *ns* not significant, *p* > 0.05; *p < 0.05; **p < 0.01; ***p < 0.001; ****p < 0.0001.

One day after infection, the chickens began to exhibit signs of depression, such as closed eyes, retracted necks, drooping wings, and reduced feed intake. Bloody stools appeared from day 3 post-infection (dpi). The Guizhou strain infection group (GSIG) exhibited markedly higher severity and duration of bloody stools compared with the Beijing strain infection group (BSIG). The BSIG exhibited a relative weight gain rate of 76.36% and feed conversion ratio of 3.7002, whereas the GSIG exhibited a relative weight gain rate of 66.41% and an FCR of 4.4132 (Figure 1A).

### Histopathological examination

Histological observation of cecal tissues revealed intact architecture in the control group (CON, Uninfected), characterized by well-organized epithelial cells and preserved villus morphology (Figure 1B). No degeneration, edema, or inflammatory infiltration was observed. By contrast, both infected groups displayed significant pathological changes (Figure 1C, D shows the groups infected with the Beijing and Guizhou strains, respectively), including intestinal wall thickening, mucosal hemorrhages (annotation A), inflammatory cell infiltration (annotation B), and villus dissolution, rupture and separation (annotation C). Numerous oocysts were also detected in the tissue sections (annotation D). Notably, the degree of intestinal villi collapse damage of Guizhou-strain-infected chickens was more serious than that of those infected with the Beijing strain.

### Pathological indices

In the Guizhou strain infection group (GSIG), the indices for the liver, spleen, and Bursa of Fabricius were 0.0400, 0.0019, and 0.0033 respectively. In the Beijing strain infection group (BSIG), the corresponding values were 0.0396, 0.0020, and 0.0029. In the control group (CON), the corresponding values were 0.0489, 0.0017, and 0.0020 (Figure 1A). These results indicated that the liver indices in both infected groups were lower than in the CON group, while the spleen and bursa of Fabricius indices were elevated. The spleen index in the GSIG group was lower than in the BSIG group, whereas the Bursa of Fabricius index and liver indices were higher in the GSIG than in the BSIG.

ELISA results further revealed that, in the BSIG, IL-17 and TNF-α levels increased significantly during the schizogony stage, with a slower rise at the sporozoite stage. In the GSIG, IL-17 expression was significantly elevated during the schizogony stage, while TNF-α levels increased rapidly during the sporozoite and peak of oocyst shedding stages (Figures 1E, F). These results showed that levels of the pro-inflammatory cytokines varied significantly across the four developmental stages (*p* < 0.05), with the GSIG showing higher expression than the BSIG at each stage (*p* < 0.05).

### Results of whole-genome resequencing

To investigate the genomic variations between strains, whole-genome resequencing was performed for the Beijing and Guizhou strains. The quality control analysis of the resequencing data is presented in Table 3. For the Beijing strain, 95.82% of the total reads were successfully mapped to the reference genome, compared with 67.03% for the Guizhou strain (Table 4). The distribution of insert sizes, calculated from the paired-end sequencing reads relative to their start and end positions on the reference genome, followed a normal distribution with a single peak (Additional files 1A, B), indicating stable and reliable sequencing performance. The peak insert size was 300 bp for the Beijing strain (Additional file 1A) and 250 bp for the Guizhou strain (Additional file 1B).

**Table 3:** **Quality assessment metrics for sequencing data**

Sample	Clean paired reads	GC (%)	Q30 (%)
BJ	6848979	41%	98.55%
GZ	16136206	49%	97.13%

BJ: *Eimeria tenella* (Beijing strain), GZ: *E. tenella* (Guizhou strain)

**Table 4:** **Statistics of read alignment with reference genome**

Sample ID	Total reads	Multiple aligned reads	Unaligned reads	Uniquely aligned reads	Overall alignment rate
BJ	27094754	5841850 (21.5608%)	1132684 (4.18046%)	20120220 (74.2587%)	95.82%
GZ	32272412	5194058 (16.0944%)	10640688 (32.9715%)	16437666 (50.9341%)	67.03%

BJ: *Eimeria tenella* (Beijing strain), GZ: *E. tenella* (Guizhou strain)

A total of 3645 and 28,690 differential SNPs were identified in the Beijing and Guizhou strains of *E. tenella*. Additionally, 391 and 3098 differential InDels were detected in the Beijing and Guizhou strains, respectively. Most SNPs of inversion type and conversion type were located in the promoter region (Additional file 1C), and the difference InDels was mainly short fragments (0–15 bp, 97.24%), including 340 insertion types and 385 deletion types (Additional file 1D).

Among the membrane protein-related coding genes (Table 5), 45 missense mutations were identified within the differential SNPs, including those in *SAG15*, *SAG13*, *RON2*, and *SAG5*, which resulted in amino acid substitutions in membrane protein. A total of 92 SNPs were located in promoter regions, including those associated with *SAG17*, *SAG15*, *RON2*, and *SAG23*. Within the differential InDels, seven missense mutations were detected, leading to frameshift mutations in membrane protein, while another seven were located in promoter regions, including members of the *SAGs*. Therefore, the two strains were significant differentiation characteristics in genetic polymorphism (Additional files 1E, F).

**Table 5:** **Genetic variation in key membrane protein genes**

Mutation types	Gene ID	BJ	GZ	Protein name	Codon mutation
SNP missense	ETH_00013150	0;0	0;1	SAG family member (SAG15)	c.130G>T
	ETH_00013178	0;0	0;1	SAG family member (SAG13)	c.27C>G
	ETH_00012760	0;0	0;1	Rhoptry neck protein 2 (RON2)	c.2516C>G
	ETH_00034960	0;0	0;1	SAG family member (SAG5)	c.468C>A
	ETH_00008720	0;0	0;1	SAG family member	c.29A>T
SNP promoter	ETH_00013130	0;0	0;1	SAG family member (SAG17)	c.-1988A>T
	ETH_00013135	0;0	0;1	SAG family member	c.-1235C>T
	ETH_00013150	0;0	0;1	SAG family member (SAG15)	c.-1205 T>G
	ETH_00012760	0;1	0;0	Rhoptry neck protein 2 (RON2)	c.-150A>G
	ETH_00035010	0;0	0;1	SAG family member	c.-1223C>A
	ETH_00008670	0;0	0;1	SAG family member (SAG23)	c.-729A>G
	ETH_00008670	0;0	0;1	SAG family member (SAG23)	c.-968A>C
	ETH_00008670	0;0	0;1	SAG family member (SAG23)	c.-970A>T
	ETH_00008670	0;0	0;1	SAG family member (SAG23)	c.-1023G>T
	ETH_00008670	0;0	0;1	SAG family member (SAG23)	c.-1172C>A
	ETH_00008670	0;0	0;1	SAG family member (SAG23)	c.-1598G>C
	ETH_00008685	0;0	0;1	SAG family member	c.-1833T>C
	ETH_00008685	0;0	0;1	SAG family member	c.-1501T>A
	ETH_00008685	0;0	0;1	SAG family member	c.-1061A>C
	ETH_00008685	0;0	0;1	SAG family member	c.-717T>C
	ETH_00008685	0;0	0;1	SAG family member	c.-481T>A
	ETH_00026045	0;1	0;0	SAG family member	c.-1228T>C
	ETH_00026045	0;0	0;1	SAG family member	c.-915A>C
	ETH_00026045	0;1	0;0	SAG family member	c.-673C>A
	ETH_00023375	0;0	0;1	SAG family member	c.-1835G>C
InDel missense	–	–	–	–	–
InDel Promoter	ETH_00013135	0;0	0;1	SAG family member	c.-112_-111insTCCT
	ETH_00023375	0;0	0;1	SAG family member	c.-1750_-1749delGC

BJ: *Eimeria tenella* (Beijing strain), GZ: *E. tenella* (Guizhou strain)

Variant type: wild‑type (0; 0), heterozygous (0; 1), homozygous (1; 1)

In the Beijing strain of *E. tenella*, SNPs and InDels were predominantly of the wild-type genotype (0; 0) accounted for 94%, indicating high genomic conservation. By contrast, heterozygous variant sites (0; 1) accounted for 96.62% of the Guizhou strain of *E. tenella*, which was significantly higher than for the Beijing strain (*p* < 0.01). Moreover, among the variant loci of the Guizhou strain, a larger proportion was located in promoter regions (64.19%) compared with the Beijing strain (2.67%), indicating more active genetic regulation of the Guizhou strain.

**Figure 2:**
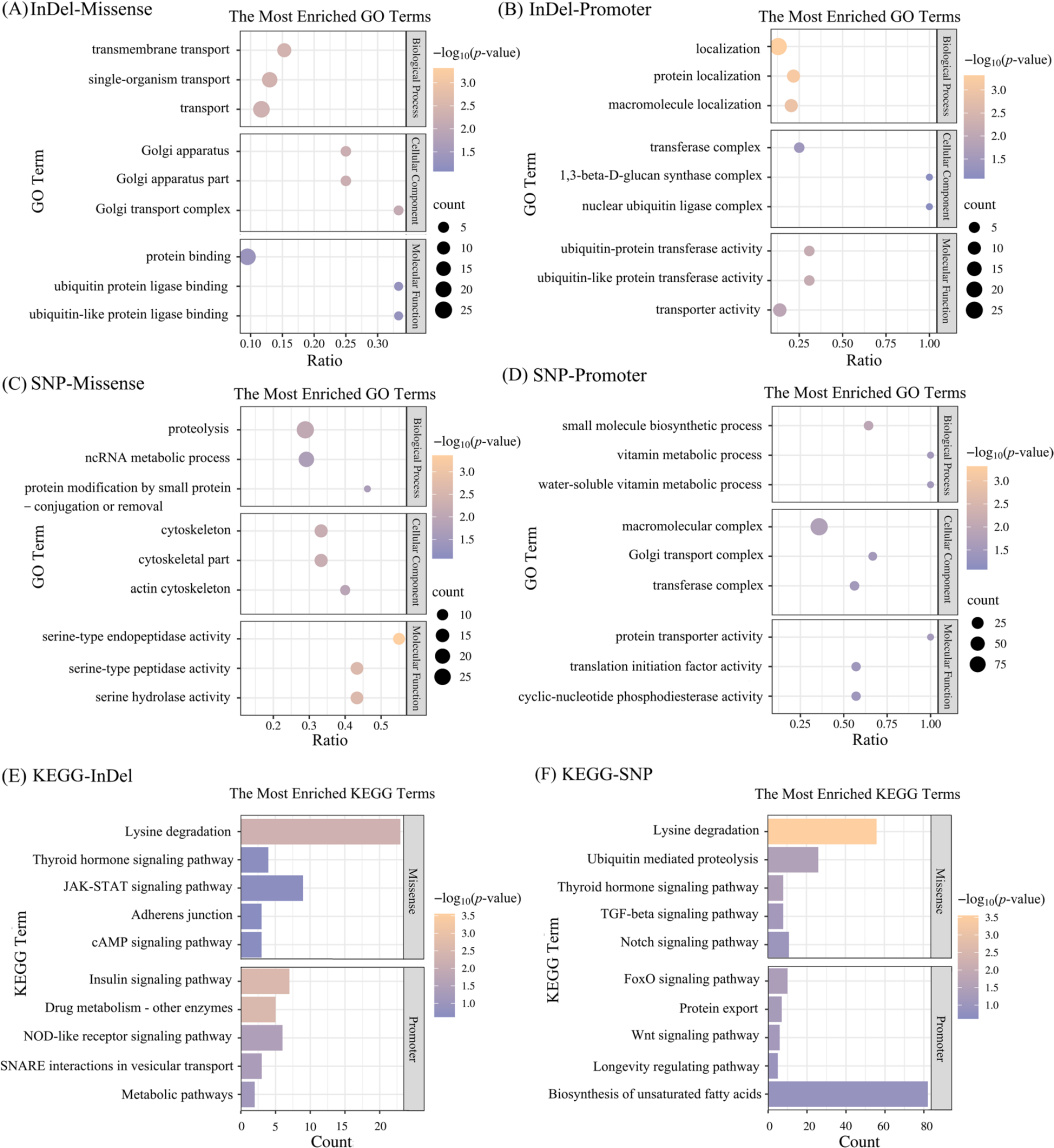
**Whole-genome resequencing results.** **A**–**D** GO enrichment bubble plots of missense and promoter-associated SNPs and InDels. **E**, **F** KEGG enrichment bar plots of missense and promoter-associated SNPs and InDels.

Gene Ontology (GO) analysis showed that genes harboring missense mutations caused by insertion or deletion events (differential InDels) between the Guizhou and Beijing strains were enriched in biological functions related to transmembrane transport and Golgi-apparatus-related activities (Figure 2A). Genes affected by promoter-located differential InDels were enriched in localization, protein localization, macromolecule localization, transferase complex, ubiquitin-protein transferase activity, ubiquitin-like protein transferase activity, and transporter activity (Figure 2B). Genes with missense mutations caused by differential SNPs were enriched in biological functions including proteolysis, cytoskeleton, and serine-type peptidase activity (Figure 2C). The genes of differential SNPs located in promoter regions were enriched in small molecule biosynthetic process, vitamin metabolic process, water-soluble vitamin metabolic process, macromolecular complex, Golgi transport complex, transferase complex, protein transporter activity, translation initiation factor activity, and cyclic-nucleotide phosphodiesterase activity (Figure 2D). Overall, differential InDels and SNPs were associated with enrichment in pathways related to protein processing, transport, and metabolic functions.

Kyoto Encyclopedia of Genes and Genomes (KEGG) analysis showed that genes harboring missense mutations caused by differential InDels were enriched in pathways related to lysine degradation, thyroid hormone signaling, JAK–STAT signaling, Adherens junction, and cyclic adenosine monophosphate (cAMP) signaling pathway. Genes affected by promoter-located differential InDels were significantly enriched in pathways associated with metabolic regulation, adaptation, material transport, and immune-related signaling, including the insulin signaling pathway, drug metabolism-other enzymes, nucleotide oligomerization domain (NOD)-like receptor signaling pathway, soluble *N*-ethylmaleimide-sensitive factor attachment protein receptor (SNARE) interactions in vesicular transport, and general metabolic pathways (Figure 2E). Genes with missense mutations caused by differential SNPs were mainly enriched in pathways such as the lysine degradation, ubiquitin mediated proteolysis, transforming growth factor (TGF)-beta signaling, and Notch signaling, as well as other pathways related to cell growth, differentiation, and protein turnover. Genes with promoter-located differential SNPs were significantly enriched in pathways including the FoxO signaling pathway, protein export, Wnt signaling pathway, longevity regulating pathway, and biosynthesis of unsaturated fatty acids (Figure 2F).

### Results of transcriptome analysis

**Figure 3:**
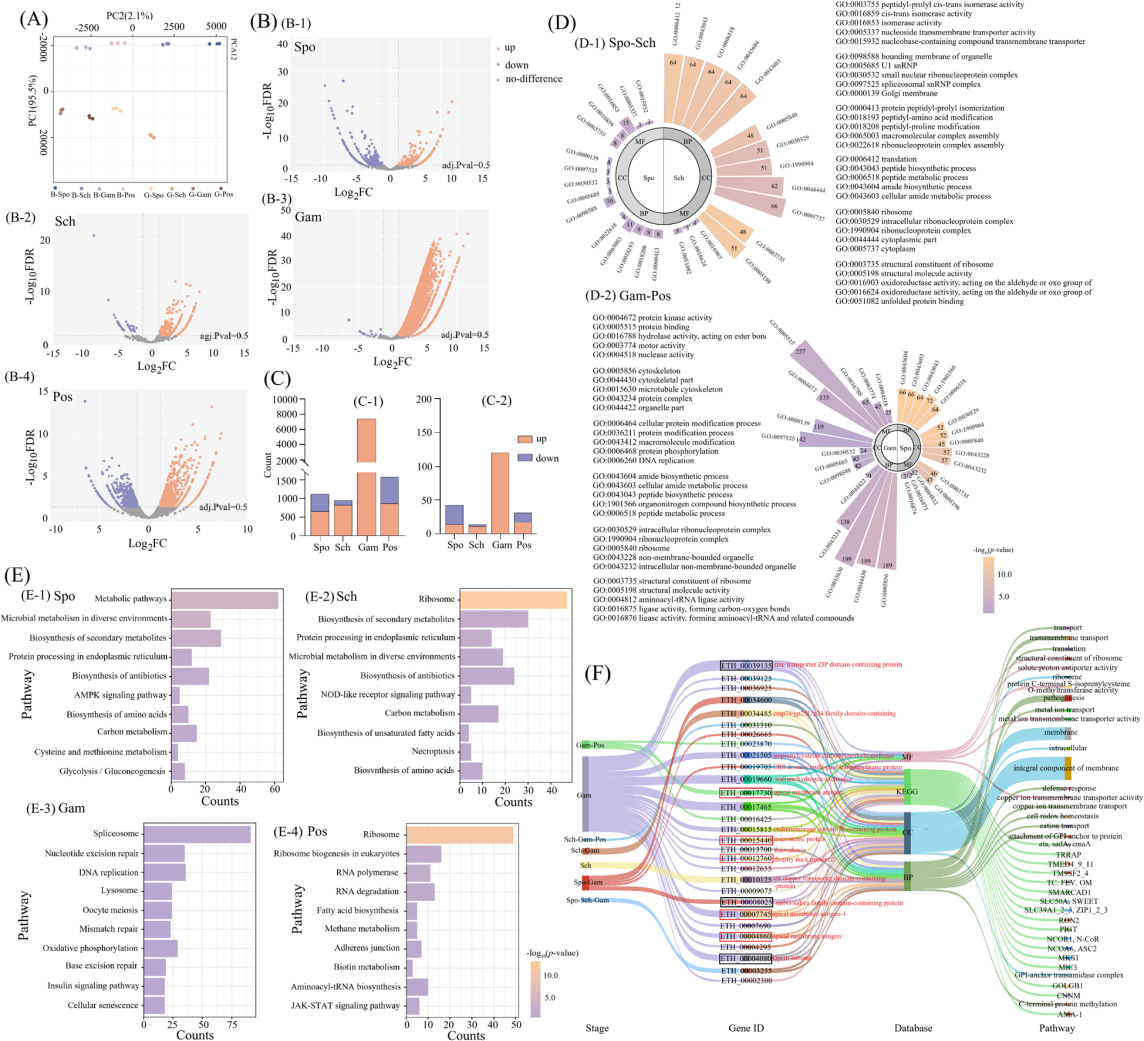
**Results of transcriptome analysis.** **A** Principal component analysis (PCA) of transcriptome profiles. **B** Volcano plots of differentially expressed genes (DEGs) at the sporozoite, schizogony, gametocyte, and peak oocyst-shedding stages (*orange*, upregulated; *blue*, downregulated). **C** Stage-specific expression of membrane protein-related genes. **D** GO enrichment of DEGs between strains across stages. **E** KEGG enrichment of DEGs between strains across stages (dot size, DEG count; color, adjusted *p*-value). **F** Sankey plot showing hierarchical associations of membrane-protein-related DEGs across stages, genes, databases, and pathways (node width, gene count; edge width, association strength).

Quality control analysis of the transcriptome sequencing data is presented in Table 6. The within-group variation was small, whereas the distance between groups was relatively large, indicating substantial overall differences between the Beijing and Guizhou strains (Figure 3A). A total of 7795 genes showed significant expression changes during the developmental process of the Guizhou strain compared with the Beijing strain. These differentially expressed genes, including membrane protein-related genes, exhibited stage-specific expression patterns (Figure 3B).

**Table 6:** **Quality assessment of sequencing samples**

Sample	Clean paired reads	GC (%)	Q30 (%)
B-Spo1	20471768	51%	96.11%
B-Spo2	21040398	50%	96.31%
B-Spo3	20351225	51%	96.45%
B-Sch1	20394961	51%	96.35%
B-Sch2	20631796	51%	95.67%
B-Sch3	21305641	50%	95.76%
B-Gam1	20870076	52%	94.88%
B-Gam2	21649164	53%	94.47%
B-Gam3	20930714	53%	95.00%
B-Pos1	20526412	51%	96.01%
B-Pos2	20522472	50%	96.20%
B-Pos3	20362530	50%	96.11%
G-Spo1	19408912	51%	96.50%
G-Spo2	20160958	51%	96.48%
G-Spo3	17603067	51%	96.63%
G-Sch1	18657695	52%	96.17%
G-Sch2	22725624	52%	96.72%
G-Sch3	17251116	52%	96.28%
G-Gam1	23666434	52%	96.82%
G-Gam2	18493220	52%	96.27%
G-Gam3	18918813	52%	96.36%
G-Pos1	18821153	52%	96.30%
G-Pos2	22051644	51%	96.19%
G-Pos3	21550831	51%	96.24%

B: *Eimeria tenella* (Beijing strain), G: *E. tenella* (Guizhou strain), Spo: sporozoite stage, Sch: schizogony stage, Gam: gametogony stage, Pos: peak of oocyst shedding

Membrane protein gene families associated with parasite invasion, such as *SAG*, *RON*, *AMA*, and *MIC*, showed marked expression differences between the two strains (Figure 3B). Figure 3C-1 illustrates the dynamic changes in the number of differentially expressed genes across the four developmental stages. Overall, compared with the Beijing strain, the Guizhou strain exhibited a significantly higher number of upregulated genes than downregulated genes (*p* < 0.05). Figure 3C-2 further depicts the dynamic expression patterns of membrane protein-related differentially expressed genes between the Guizhou and Beijing strains across different developmental stages. In the sporozoite stage, a total of 42 membrane-protein-related genes were differentially expressed, including 14 upregulated and 28 downregulated genes, among which 19 of the 20 SAG family DEGs were downregulated (Table 7). During the schizogony stage, 14 membrane-protein-related genes were identified, with 11 upregulated and 3 downregulated genes. In the gametocyte stage, all 120 identified membrane-protein-related genes were upregulated, including 3 AMA family genes, 20 SAG family genes, and 1 RON family gene (Table 7). During the oocyst shedding stage, 31 membrane-protein-related genes were differentially expressed, including 17 upregulated and 14 downregulated genes, of which 3 SAG family genes and 1 AMA family gene were downregulated (Table 7).

**Table 7:** **Differential genes related to membrane proteins**

Stage	Gene ID	Protein name
Spo	ETH_00013130	SAG family member (SAG17)
	ETH_00013135	SAG family member
	ETH_00013178	SAG family member (SAG13)
	ETH_00034930	SAG family member
	ETH_00034935	SAG family member
	ETH_00034945	SAG family member (SAG6)
	ETH_00034950	SAG family member (SAG7)
	ETH_00034960	SAG family member (SAG5)
	ETH_00034965	SAG family member
	ETH_00034975	SAG family member (SAG10)
	ETH_00034980	SAG family member
	ETH_00035015	SAG family member
	ETH_00035025	SAG family member
	ETH_00026045	SAG family member
	ETH_00035030	SAG family member (SAG11)
	ETH_00008670	SAG family member (SAG23)
	ETH_00008685	SAG family member
	ETH_00008700	SAG family member
	ETH_00008720	SAG family member
	ETH_00008745	SAG family member (SAG20)
Sch	–	–
Gam	ETH_00004860	Apical membrane antigen, putative (AMA)
	ETH_00013130	SAG family member (SAG17)
	ETH_00013135	SAG family member
	ETH_00013150	SAG family member (SAG15)
	ETH_00007745	Apical membrane antigen-1
	ETH_00012760	Rhoptry neck protein 2 (RON2)
	ETH_00034880	SAG family member
	ETH_00034930	SAG family member
	ETH_00034935	SAG family member
	ETH_00034945	SAG family member (SAG6)
	ETH_00034950	SAG family member (SAG7)
	ETH_00034960	SAG family member (SAG5)
	ETH_00035015	SAG family member
	ETH_00034965	SAG family member
	ETH_00034975	SAG family member (SAG10)
	ETH_00034980	SAG family member
	ETH_00035010	SAG family member
	ETH_00035025	SAG family member
	ETH_00035030	SAG family member (SAG11)
	ETH_00015440	Microneme protein (MIC)
	ETH_00008685	SAG family member
	ETH_00008700	SAG family member
	ETH_00008720	SAG family member
	ETH_00008745	SAG family member (SAG20)
	ETH_00017730	apical membrane antigen (AMA)
Pos	ETH_00035015	SAG family member
	ETH_00035025	SAG family member
	ETH_00017730	apical membrane antigen (AMA)

Spo: sporozoite stage, Sch: schizogony stage, Gam: gametogony stage, Pos: peak of oocyst shedding

GO enrichment analysis indicated that the differentially expressed genes in the sporozoite stage were mainly enriched in biological processes such as peptidyl-amino acid modification, macromolecular complex assembly, and nucleobase-containing compound transmembrane transport. In the schizogony stage, enrichment was primarily observed in translation, peptide biosynthetic process, ribosome, structural constituent of ribosome, and oxidoreductase activity (Figure 3D-1). In the gametocyte stage, genes were mainly enriched in pathways including cellular protein modification, protein phosphorylation, DNA replication, cytoskeleton, and microtubule cytoskeleton. In the peak oocyst shedding stage, the enriched biological processes were highly similar to those identified in the schizogony stage (Figure 3D-2).

**Figure 4:**
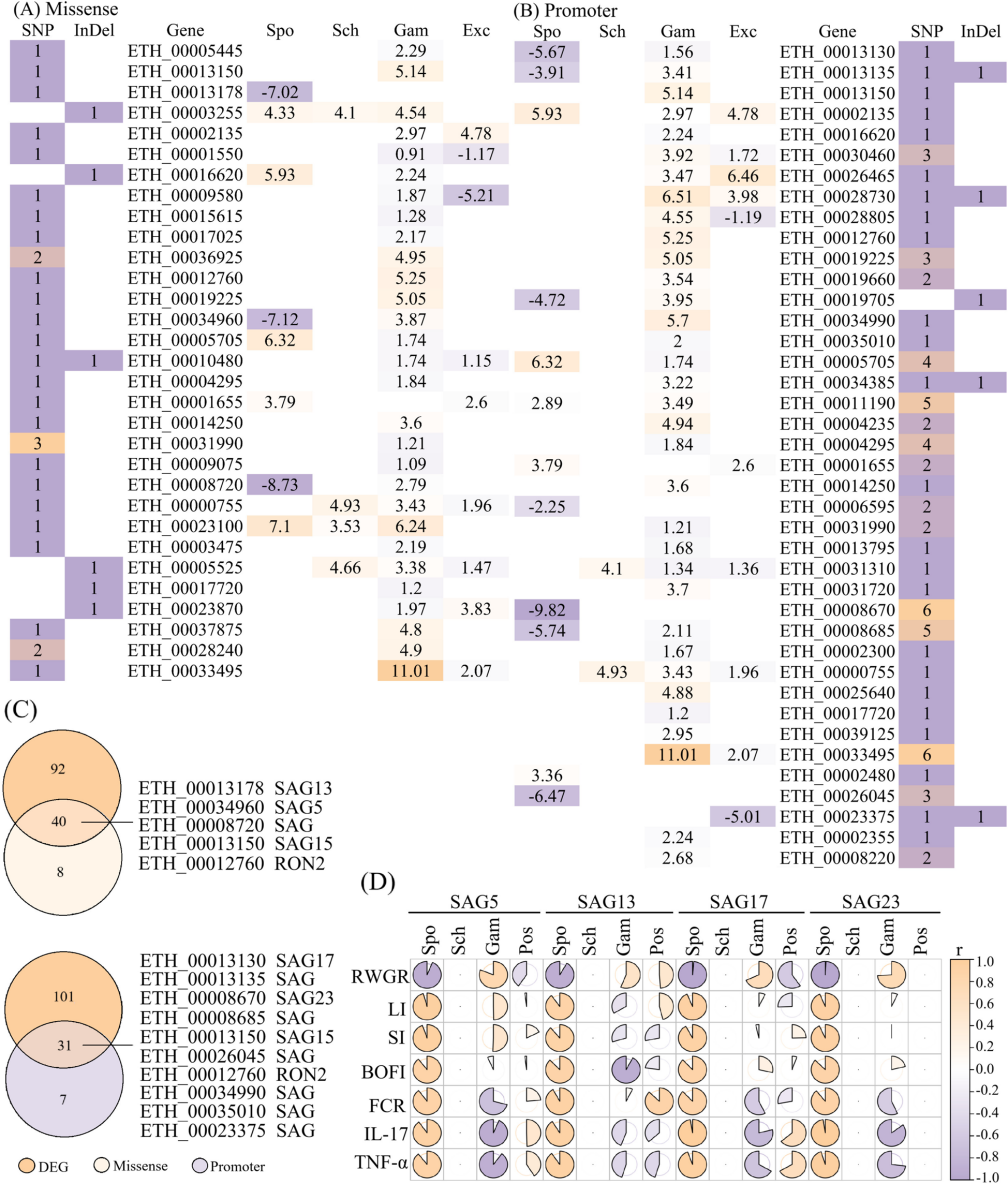
**Differential expression of key membrane protein genes and correlations with clinical phenotypes**. **A**, **B** Heatmaps of DEGs from the Beijing and Guizhou strains carrying missense or promoter-associated SNPs/indels (log10 FPKM; zeros imputed as half of the minimum). **C** Venn diagram of candidate genes (only annotated proteins shown; unannotated genes are hypothetical proteins). **D** Correlations between clinical phenotypes and stage-specific expression of candidate genes (Gene-Spo/Sch/Gam/Pos denote expression level of genes at the sporozoite, schizogony, gametocyte, and peak oocyst-shedding stages, respectively). Color indicates correlation coefficients (−1 to 1; *purple*, negative; *orange*, positive).

KEGG enrichment analysis revealed distinct stage-specific patterns of differentially expressed genes (DEGs) across the four developmental stages (Figure 3E). In the sporozoite stage, DEGs were significantly enriched (*p* < 0.05) in pathways including metabolic pathways, carbon metabolism, and glycolysis/gluconeogenesis (Figure 3E-1). Additional enriched pathways included microbial metabolism in diverse environments, biosynthesis of secondary metabolites, protein processing in the endoplasmic reticulum, 5′-adenosine monophosphate-activated protein kinase (AMPK) signaling pathway, cysteine and methionine metabolism, and biosynthesis of antibiotics. In the schizogony stage, DEGs were primarily enriched in pathways related to ribosome, NOD-like receptor signaling pathway, necroptosis, and biosynthesis of unsaturated fatty acids (Figure 3E-2). No significantly enriched pathways were detected in the gametocyte stage (*p* > 0.05; Figure 3E-3). At the peak of oocyst shedding, DEGs were mainly enriched in pathways associated with ribosome, ribosome biogenesis in eukaryotes, RNA polymerase, and RNA degradation (Figure 3E-4).

Key invasion-related genes, including AMA family members (ETH_00007745, ETH_00017730, ETH_00004860), RON2 (ETH_00012760), and MIC-related genes (ETH_00015440), were mapped to pathways associated with parasite entry and invasion processes. Additional differentially expressed genes included zinc transporter ZIP domain-containing protein (ETH_00039135), mtN3/saliva family domain-containing protein (ETH_00008025), and Gpi16 subunit (ETH_00004080).

The hierarchical associations and gene flow distribution of membrane protein-related differentially expressed genes (DEGs) between the Beijing and Guizhou strains across KEGG pathways and GO functional categories are shown in Figure 3F. Membrane-protein-related genes were primarily enriched in membrane-associated structures (integral component of membrane, membrane) and pathogenesis. Apical membrane antigen 1 (ETH_00007745) was mapped to the AMA-1 pathway, and rhoptry neck protein 2 (ETH_00012760) was mapped to the RON2 pathway. Microneme protein (ETH_00015440) was mapped to the MIC3 pathway. The GPI transamidase subunit Gpi16 (ETH_00004080) was mapped to the PIGT pathway and was enriched in GPI anchor–protein linkage processes. Differences in enrichment patterns between the Beijing and Guizhou strains are shown in Figure 3F.

### Screening of key membrane-protein genes

Whole-genome sequencing (WGS) and RNA-sequencing (RNA-seq) data were jointly analyzed to screen for membrane protein genes across the four developmental stages. A total of 44 differentially expressed membrane protein–related genes were identified, and these genes harbored genetic variants (Figures 4A, B). Notably, *SAG17* and *SAG23* contained one and six SNPs, respectively, all of which were located within promoter regions (Figure 4B) and therefore did not affect the coding sequences or predicted transmembrane domains of the corresponding proteins. Among these, 13 genes carried both missense and promoter variants, including SAG and RON family members such as *SAG13*, *SAG5*, *SAG15*, *RON2*, *SAG17*, and *SAG23* (Figure 4C).

Pearson correlation analysis revealed that, at the sporozoite stage, all seven clinical phenotypic parameters exhibited strong correlations with the expression levels of *SAG5*, *SAG13*, *SAG17*, and *SAG23* (*r* > 0.85), with mean correlation coefficients of 0.9179, 0.9020, 0.9317, and 0.9358, respectively (Figure 4D). In contrast, no significant correlations were observed at the schizont stage, whereas only weak correlations were detected at the gametocyte stage and the peak of oocyst shedding.

**Figure 5:**
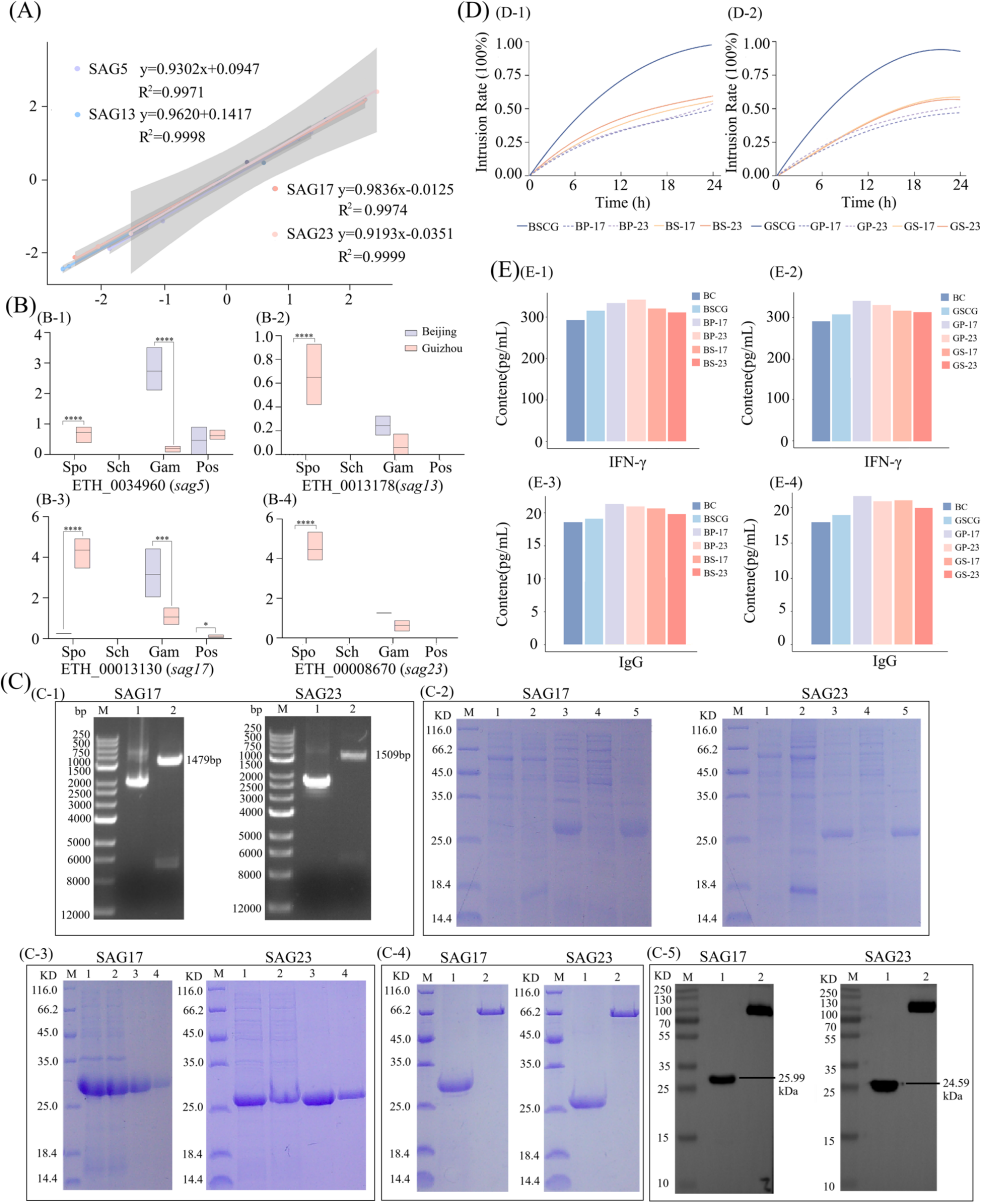
**Expression validation of the key SAG genes and in vitro intervention with recombinant SAG17/SAG23.** Results of key gene expression analysis and in vitro intervention experiments with recombinant SAG17/SAG23 proteins. **A** Linear regression analysis between qRT-PCR and RNA-seq results, with the y-axis showing Log₂(RNA-seq) and the x-axis showing Log₂(qRT-PCR). **B** Stage-specific expression of *SAG5* (ETH_00034960), *SAG13* (ETH_00013178), *SAG17* (ETH_00013130), and *SAG23* (ETH_00008670). **C-1** Restriction enzyme digestion profiles. M: DNA marker; Lane 1: plasmid; Lane 2: plasmid digested with NdeI–XbaI; **C-2** SDS-PAGE analysis of protein expression. M: protein marker; Lane 1: pCZN1 empty vector (induced); Lane 2: uninduced; Lane 3: induced; Lane 4: supernatant after cell lysis; Lane 5: pellet after cell lysis; **C-3** SDS-PAGE analysis of protein purification. M: protein marker; Lane 1: lysate; Lane 2: flow-through; Lanes 3–4: eluates; **C-4** SDS-PAGE of purified proteins. M: protein marker; Lane 1: purified protein; Lane 2: 0.5 mg/mL BSA; **C-5** Western blot analysis of purified proteins. M: protein marker; Lane 1: purified protein; Lane 2: Multitag protein. **D** Effects of recombinant SAG17 and SAG23 on invasion of *Eimeria tenella* sporozoites (Beijing versus Guizhou strains) into PCEC. **E** Effects of recombinant SAG17 and SAG23 on IFN-γ and IgG levels following sporozoite invasion in PCEC. *ns* not significant, *p* > 0.05; *p < 0.05; **p < 0.01; ***p < 0.001; ****p < 0.0001.

RT-qPCR was used to examine the expression levels of the four selected *SAG* genes (Figure 5A), and the FPKM expression profiles of *SAG5*, *SAG13*, *SAG17*, and *SAG23* across developmental stages were visualized (Figure 5B). The expression levels of these four genes in the Guizhou strain were significantly higher than those in the Beijing strain during the sporozoite stage (*p* < 0.01). Their expression levels in the Guizhou strain during the sporozoite stage were also significantly higher than those in the other developmental stages (*p* < 0.01). The expression patterns of the four *SAG* genes showed strong consistency with their RNA-seq FPKM values (*r* > 0.85). During the sporozoite stage, *SAG17* and *SAG23* were expressed at higher levels than *SAG5* and *SAG13*, and their expression showed the strongest correlation with clinical phenotypes (Figure 4).

Transcriptomic analysis revealed that differentially expressed genes at the sporozoite stage included eight members of the MIC family and 47 members of the SAG family (Table 7). Whole-genome resequencing further showed that only *SAG13*, *SAG5*, *SAG17*, and *SAG23* simultaneously harbored missense mutations and promoter-region variants (Figure 4C). qPCR validation confirmed that *SAG17* and *SAG23* exhibited the most pronounced expression levels at the sporozoite stage (Figures 5A, B). Taken together, the integration of transcriptomic profiling, genomic variation analysis, and expression validation identified *SAG17* and *SAG23* as core candidate genes underlying virulence divergence among *E. tenella* strains.

### Prokaryotic expression of recombinant SAG17 and SAG23 proteins

Both *SAG17* and *SAG23* genes were successfully cloned into pCZN1 vectors and expressed, yielding bands of the expected sizes (*SAG17*: 1479 bp; *SAG23*: 1509 bp) and proteins consistent with the predicted molecular weights (SAG17: 25.99 kDa; SAG23: 24.59 kDa). The purified recombinant SAG17 and SAG23 proteins reached >85% purity with final concentrations of 1.0 mg/mL (Figure 5C).

### In vitro protein intervention results

Treatment with recombinant SAG17 or SAG23 proteins alone significantly inhibited sporozoite invasion into host cells (Figure 5D). Compared with the BSIG group (Figure 5D-1), invasion rates in the pretreatment groups were reduced by 48.61% in BP-17 (*p* < 0.01) and 44.82% in BP-23, whereas in the synchronous treatment groups, invasion was reduced by 42.58% in BS-17 (*p* < 0.01) and 38.62% in BS-23. Compared with the GSIG group (Figure 5D-2), invasion rates in the pretreatment groups were reduced by 46.10% in GP-17 (*p* < 0.01) and 41.55% in GP-23, while reductions in the synchronous treatment groups reached 34.48% in GS-17 (*p* < 0.01) and 36.08% in GS-23.

Cytokine and immunoglobulin measurements showed differences between treatment groups following exposure to recombinant SAG17 and SAG23 proteins. When the proteins were administered prior to infection, IFN-γ and IgG levels in PCECs were higher than those observed under synchronous treatment (Figure 5E).

## Discussion

Coccidiosis caused by *E. tenella* is a major parasitic disease that severely impacts global poultry production, often resulting in cecal hemorrhage, impaired nutrient absorption, and mortality [1–4]. Environmental conditions, farming practices, and host immune backgrounds have been shown to drive long-term adaptive divergence among geographically distinct strains [45]. In this study, infection outcomes demonstrated that the Guizhou strain exhibited markedly stronger pathogenicity than the Beijing strain, consistent with field observations and ecological differences between the two regions. Geographical separation and differences in rearing conditions may have contributed to the genetic divergence observed between the strains [46].

In this study, a systematic comparison of the Beijing and Guizhou strains of *E. tenella* confirmed that *SAG17* and *SAG23* played critical roles in strain-specific virulence divergence. This finding not only expands the current understanding of the pathogenic mechanisms of *E. tenella* but also provides a solid molecular basis for cross-regional precision control strategies, including vaccine development and novel drug target identification.

### Stronger inflammatory responses may contribute to the higher pathogenicity of the Guizhou strain

The Guizhou strain induced more severe clinical symptoms and tissue lesions than the Beijing strain (Figure 1). Cytokine analysis revealed that infection with the Guizhou strain induced higher levels of IL-17 and TNF-α (Figure 1E, F), with dynamic patterns distinct from those of the Beijing strain, suggesting that it may trigger a more intense inflammatory response in the host. Previous studies have demonstrated that cytokines such as IFN-γ, IL-10, IL-17, and TNF-α were closely associated with tissue damage and immune balance during coccidial infection [21, 22, 47, 48]. Therefore, the enhanced pathogenicity of the Guizhou strain is likely related to its ability to elicit a stronger inflammatory response.

### Variations in membrane-protein-related genes as a potential molecular basis of strain-specific virulence divergence

Whole-genome resequencing revealed substantial differences in membrane-protein-related genes between the Beijing and Guizhou strains (Table 5), with the Guizhou strain presenting more nonsynonymous substitutions and promoter variants in key invasion-associated families such as SAGs and RONs. Functional annotation indicated that these variants were associated with transmembrane transport, protein processing, cytoskeletal regulation, and immune-related pathways (Figure 2). Although further functional verification is needed, such genetic variations may influence the expression or regulation of membrane proteins, potentially contributing to phenotypic divergence in virulence.

### Stage-dependent transcriptional regulation of membrane proteins may enhance parasite invasion capacity

Transcriptome analysis demonstrated that membrane-protein-related genes exhibited dynamic differential expression across developmental stages, consistent with stage-specific transcriptional features observed in *Toxoplasma gondii* and *Plasmodium falciparum* [49, 50]. Such stage-dependent regulation is considered to facilitate a balance between parasite development and pathogenicity within the host. In the sporozoite stage, SAGs of the Guizhou strain showed a marked trend of high expression (Figure 5B), suggesting stronger capacity for host recognition and adhesion [51]. Previous studies demonstrated that SAG, RON, AMA, and MIC proteins represented core molecules mediating apicomplexan parasite invasion [52, 53]. The differential expression and functional regulation of these proteins were likely critical determinants of strain-specific virulence divergence. In this study, differentially expressed genes were simultaneously enriched in pathways related to protein modification, organelle assembly, and energy metabolism. These observations led us to hypothesize that the Guizhou strain may exhibit enhanced invasive capacity mediated by dynamic transcriptional regulation of membrane proteins.

### Differential expression of *SAG* genes drives host recognition, potentially with immune regulatory functions

SAGs are the earliest membrane proteins exposed to the host immune system and are directly involved in host cell recognition, adhesion, and invasion [11, 54]. SAG proteins are anchored to the parasite surface via glycosylphosphatidylinositol (GPI) and function in adhesion, immune recognition, and immune evasion [16]. Previous studies have reported that certain pathogen-secreted membrane proteins could be inserted into host-cell membranes as integral components, thereby altering the physicochemical properties of the host membrane and facilitating parasite colonization and proliferation [55]. Variations in membrane-integrated proteins not only determine the efficiency of pathogen recognition and binding but also potentially influence host specificity and tissue tropism [56].

The Guizhou strain exhibited markedly higher expression levels of *SAG* genes compared with the Beijing strain (*p* < 0.05), accompanied by promoter mutations and InDel variations, indicating that both transcriptional regulation and genetic alterations contribute to virulence differentiation. The promoter region, as a key *cis*-regulatory element responsible for transcription initiation, has been shown to modulate gene transcriptional output through sequence variations that alter the affinity and occupancy of transcription factor binding sites, thereby leading to either upregulation or downregulation of gene expression [57, 58]. Accordingly, the genetic variations identified in the upstream promoter regions of SAG17 and SAG23 in the Guizhou strain were likely to enhance their transcriptional activity, which may represent one of the major reasons underlying their significantly higher expression levels compared with those observed in the Beijing strain. The correlations of *SAG5*, *SAG13*, *SAG17*, and *SAG23* subtypes with host growth performance and immune organ indices, together with the immunomodulatory effects of recombinant SAG17 and SAG23 proteins on IFN-γ and IgG production, suggest that SAGs function not only as virulence factors but also as potential vaccine and therapeutic targets. These findings are consistent with observations in other apicomplexan parasites, in which the SAG/AMA–RON complex has been demonstrated to play an essential role in host-cell invasion [53]. Thus, the elevated expression of SAGs in the Guizhou strain may have facilitated immune evasion and sustained infection by interfering with immune cell activation and cytokine secretion [59].

The enhanced pathogenicity of the Guizhou strain was likely driven by the combined effects of elevated *SAG17* and *SAG23* expression and genetic variations, which conferred potential functional advantages and a potential dual strategy characterized by “high adhesion and possibly enhanced immune evasion” within the host. This finding not only provided molecular evidence for explaining the mechanisms underlying geographical strain divergence but also highlighted *SAG* genes as promising targets for vaccine and drug development.

### Evolutionary divergence between geographically distinct *E. tenella* strains

The lower genome alignment rate observed for the Guizhou strain primarily reflects its pronounced evolutionary divergence from the reference genome (*E. tenella* Houghton strain), rather than limitations in sequencing quality. Compared with the Beijing strain, the Guizhou strain has accumulated substantially more nonsynonymous mutations in both promoter and coding regions of several virulence-associated genes (e.g., *SAG15*, *SAG17*, and *RON2*), accompanied by clear signatures of balancing selection [60]. The Guizhou strain is a clinical isolate originating from Guizhou Province and has undergone long-term adaptation to local host populations and ecological conditions, resulting in the accumulation of extensive SNPs, InDels, and larger insertion/deletion events. These evolutionary changes increase sequence divergence from the reference genome and consequently reduce alignment efficiency. In contrast, the Beijing strain has undergone prolonged laboratory passaging and purification, maintaining higher genomic conservation and thus exhibiting a higher alignment rate.

Importantly, the sequencing quality for the Guizhou strain was robust. Quality control metrics showed a Q30 value of 97.13% and approximately 1.6 × 10^7^ clean reads, indicating sufficient sequencing depth and data quality for downstream analyses. Therefore, the observed alignment rate difference mainly reflects biological divergence between geographically distinct strains rather than technical artifacts.

The lower alignment rate may partially affect downstream analyses, for example by reducing the detection sensitivity of variants in highly divergent regions or limiting annotation accuracy based on a reference genome. To minimize such effects, stringent quality control measures were applied during variant calling. SNP detection was performed using both SAMtools mpileup and VarScan, with strict filtering criteria (-min-coverage 20, -min-reads 25, -min-freq-for-hom 0.75) to exclude low-confidence and poorly supported variants, thereby ensuring the robustness and reliability of the results.

Although the Guizhou strain was associated with higher pathogenicity and more severe host tissue damage, significantly fewer oocysts were produced than by the Beijing strain (Figure 1A, Additional file 4). This pattern might have reflected a trade-off whereby enhanced invasive capacity and acute virulence were achieved at the expense of reduced parasite fecundity and/or oocyst shedding efficiency, which is consistent with previous studies [61, 62]. In response to this discrepancy in oocyst output, ongoing work in our group has been initiated and is currently addressing this phenomenon by examining key genes expressed during the gametocyte stage and the oocyst egress phase. The study results will be presented in our subsequent research article.

Nevertheless, certain limitations remain: only two strains (Beijing and Guizhou) were compared, and functional validation was restricted to in vitro assays without extensive multistrain or in vivo confirmation. Future studies should therefore incorporate a broader range of geographical isolates and animal infection models to further elucidate the molecular interactions between *SAG17*/*SAG23*, host receptors, and downstream signaling pathways, thereby strengthening the foundation for rational vaccine design and therapeutic development.

## Conclusions

This study compared the Beijing and Guizhou strains of *E. tenella* and revealed significant virulence divergence between the two geographical isolates, identifying SAG17 and SAG23 as key molecules driving strain-specific differences. In the Guizhou strain, elevated expression of *SAG* genes, together with promoter and coding sequence variations, enhanced parasite adhesion to host cells and inflammation regulation capacity. In vitro functional assays further demonstrated that recombinant SAG17 and SAG23 proteins effectively blocked sporozoite invasion and activated host immune responses, highlighting their potential as targets for vaccine and drug development. Collectively, this study provides preliminary molecular evidence of the early-stage mechanisms underlying the divergence of virulence among different strains of *E. tenella*, which could inform the precision prevention and control of chicken coccidiosis, as well as the development of novel anticoccidial strategies.

## Supplementary Information

**Additional file 1: Analysis of variation characteristics in resequencing.** It provides supplementary visual support for the whole-genome resequencing results in Section "Results of whole-genome resequencing".

**Additional file 2: Results of immunofluorescence identification of PCEC and sporozoite invasion into PCEC.** Provides supplementary support for cell purity identification and in vitro invasion assays in Section "In vitro protein intervention results".

**Additional file 3: Original images of expression and detection results of recombinant SAG17 and SAG23 proteins.** Provides raw data support for the protein expression results in Section "Prokaryotic expression of recombinant SAG17 and SAG23 proteins".

**Additional file 4: Changes in OPG from 1 to 8 days post *Eimeria tenella* infection.**

## Data Availability

Raw transcriptome data have been deposited in the NCBI BioProject database under accession nos. PRJNA1163523 (Beijing strain) and PRJNA1327252 (Guizhou strain) [63, 64]. Raw whole-genome resequencing data have been deposited in the NCBI Sequence Read Archive (SRA) under accession nos. SRR35396008 (Beijing strain) and SRR35396007 (Guizhou strain) [65, 66].
