# Supplementary material for: Integrative comparative genomics and transcriptomics reveal key roles of SAG17 and SAG23 in early-stage virulence divergence of Eimeria tenella
Source: Vet Res. 2026 Apr 28;57:86. doi: 10.1186/s13567-026-01730-0 (PMC13214288; doi:10.1186/s13567-026-01730-0)
Supplement: Supplementary file 1 — Additional file 1: Analysis of variation characteristics in resequencing. It provides supplementary visual support for the whole-genome resequencing results in Section "Results of whole-genome resequencing". [file 13567_2026_1730_MOESM1_ESM.docx]

**Title:** Integrative comparative genomics and transcriptomics reveal key roles of *SAG17* and *SAG23* in early-stage virulence divergence of *Eimeria tenella*

**Authors:** Y. He, X. Wan, X. Wang, Y. Chen, D. He, Y. Yu, S. Dong, M. Wu, L. Cao, B. Wang


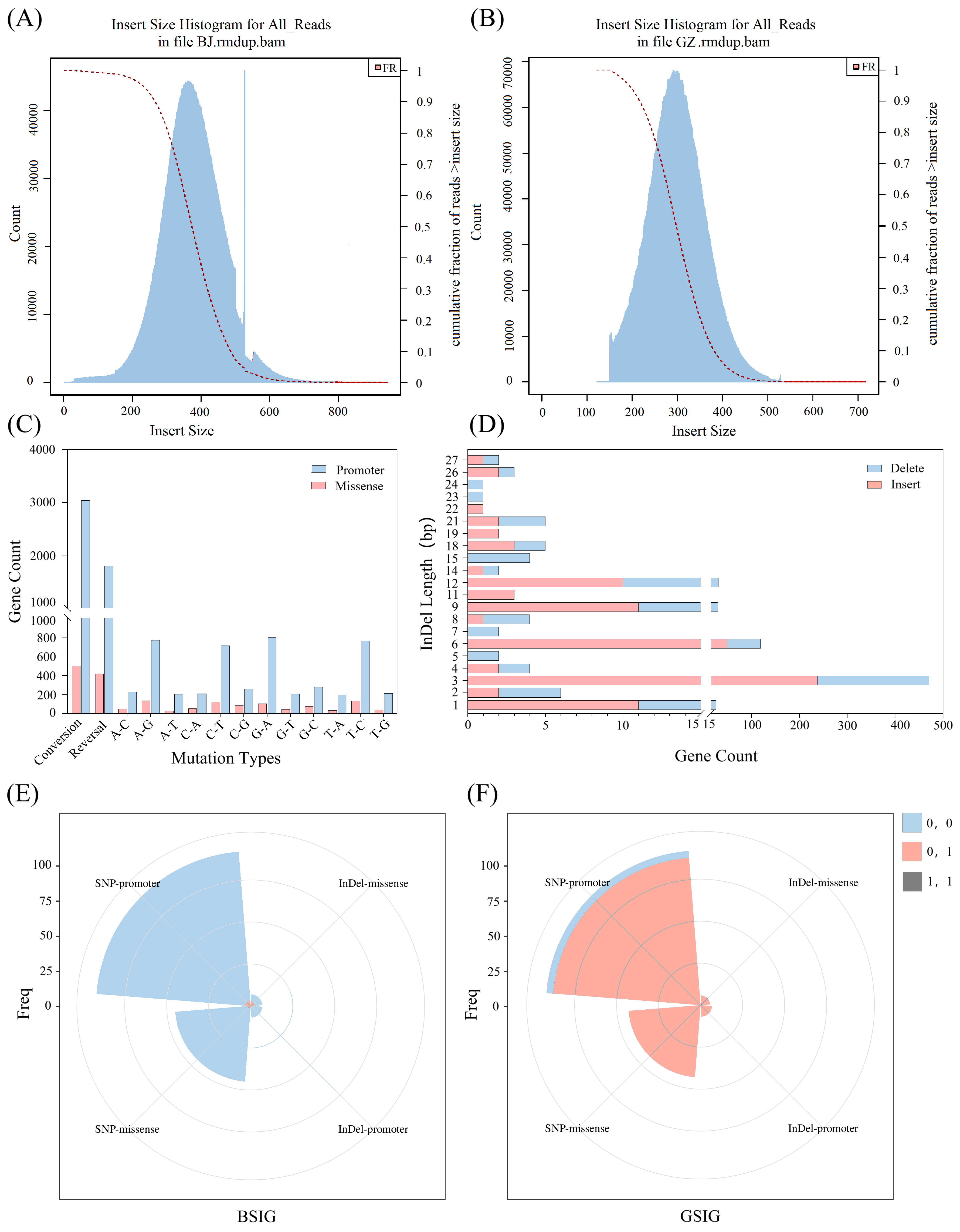


**Additional file 1. Analysis of variation characteristics in resequencing. (A–B)** Distribution of insert sizes in Beijing and Guizhou strains. **(C)** SNP classifications annotated as missense or located in promoter regions. **(D)** Length distribution of InDels. **(E–F)** Genetic variation patterns in membrane protein-related genes of *Eimeria tenella* Beijing and Guizhou strains, showing wild type (0; 0), heterozygous (0; 1), and homozygous (1; 1) variants.
