# Supplementary material for: Integrative comparative genomics and transcriptomics reveal key roles of SAG17 and SAG23 in early-stage virulence divergence of Eimeria tenella
Source: Vet Res. 2026 Apr 28;57:86. doi: 10.1186/s13567-026-01730-0 (PMC13214288; doi:10.1186/s13567-026-01730-0)
Supplement: Supplementary file 2 — Additional file 2: Results of immunofluorescence identification of PCEC and sporozoite invasion into PCEC. Provides supplementary support for cell purity identification and in vitro invasion assays in Section "In vitro protein intervention results". [file 13567_2026_1730_MOESM2_ESM.docx]

**Title:** Integrative comparative genomics and transcriptomics reveal key roles of *SAG17* and *SAG23* in early-stage virulence divergence of *Eimeria tenella*

**Authors:** Y. He, X. Wan, X. Wang, Y. Chen, D. He, Y. Yu, S. Dong, M. Wu, L. Cao, B. Wang


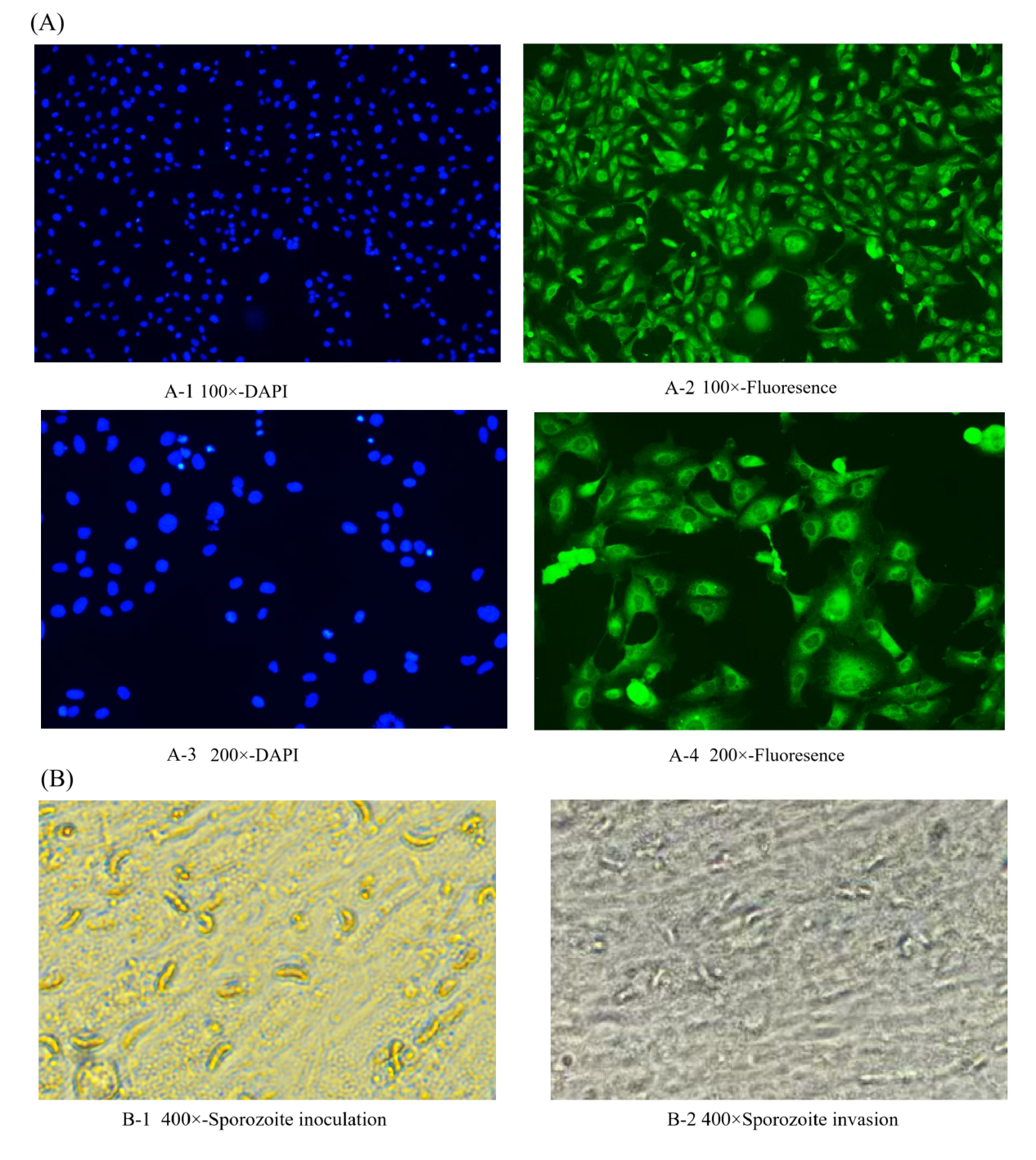


**Additional file 2. Results of immunofluorescence identification of PCEC and sporozoite invasion into PCEC.** **(A)** Identification of primary cecal epithelial cells. **(B)** Extraction of *Eimeria tenella* sporozoites and cell infection diagram.
