# Supplementary material for: Integrative comparative genomics and transcriptomics reveal key roles of SAG17 and SAG23 in early-stage virulence divergence of Eimeria tenella
Source: Vet Res. 2026 Apr 28;57:86. doi: 10.1186/s13567-026-01730-0 (PMC13214288; doi:10.1186/s13567-026-01730-0)
Supplement: Supplementary file 3 — Additional file 3: Original images of expression and detection results of recombinant SAG17 and SAG23 proteins. Provides raw data support for the protein expression results in Section "Prokaryotic expression of recombinant SAG17 and SAG23 proteins". [file 13567_2026_1730_MOESM3_ESM.docx]

**Title:** Integrative comparative genomics and transcriptomics reveal key roles of *SAG17* and *SAG23* in early-stage virulence divergence of *Eimeria tenella*

**Authors:** Y. He, X. Wan, X. Wang, Y. Chen, D. He, Y. Yu, S. Dong, M. Wu, L. Cao, B. Wang


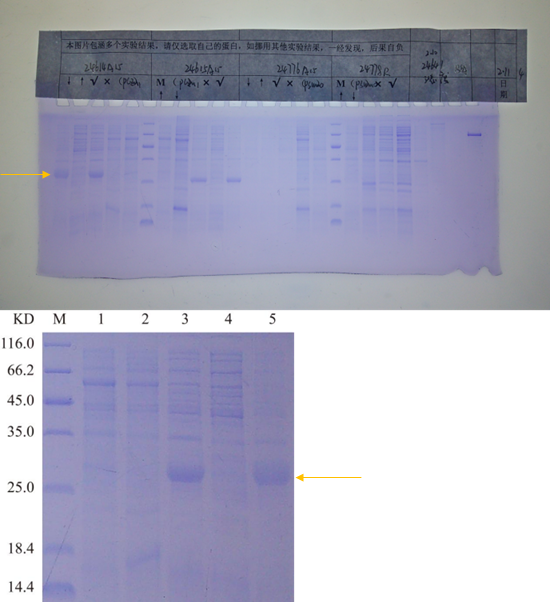


**Figure 1**. Original images of the expression and detection results of recombinant SAG17 and SAG23 proteins, among which the original image of recombinant protein SAG17 corresponds to Fig. 5C-2.


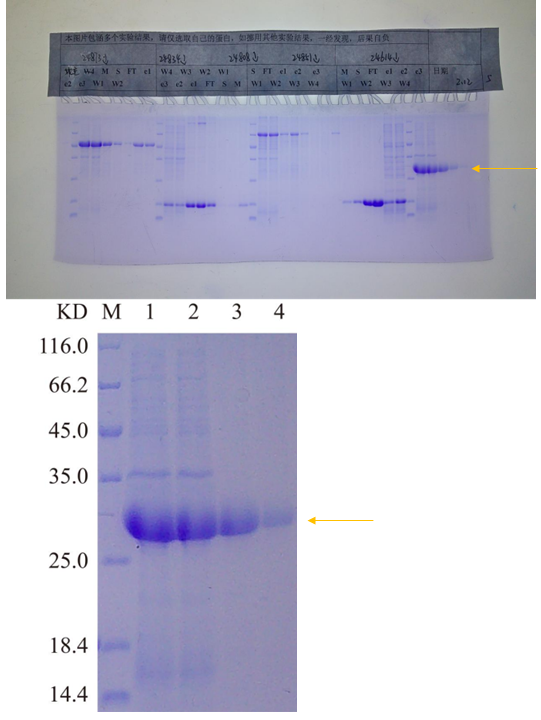


**Figure 2**. Original images of the expression and detection results of recombinant SAG17 and SAG23 proteins, among which the original image of recombinant protein SAG17 corresponds to Fig. 5C-3.


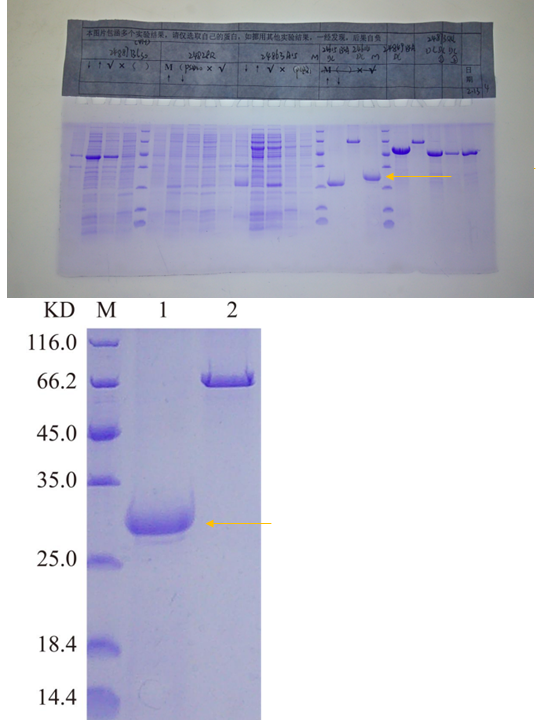


**Figure 3**. Original images of the expression and detection results of recombinant SAG17 and SAG23 proteins, among which the original image of recombinant protein SAG17 corresponds to Fig. 5C-4.


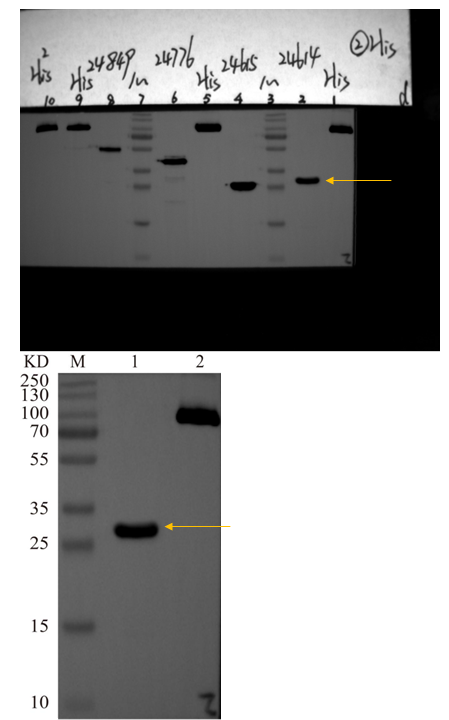


**Figure 4**. Original images of the expression and detection results of recombinant SAG17 and SAG23 proteins, among which the original image of recombinant protein SAG17 corresponds to Fig. 5C-5.


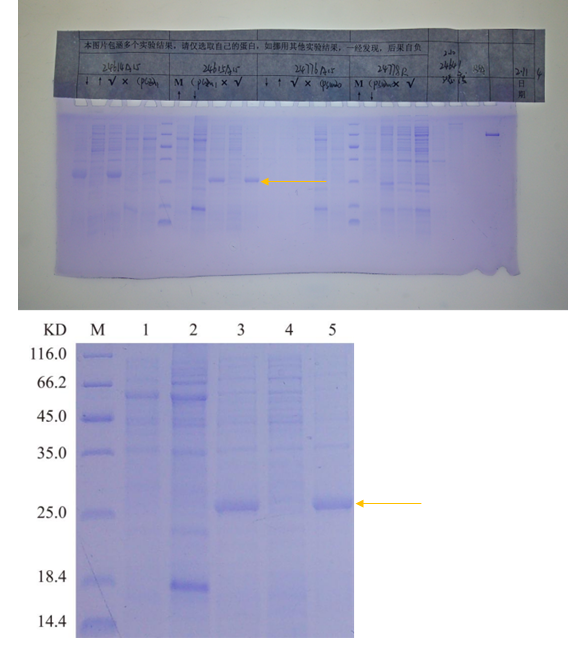


**Figure 5**. Original images of the expression and detection results of recombinant SAG17 and SAG23 proteins, among which the original image of recombinant protein SAG23 corresponds to Fig. 5C-2.


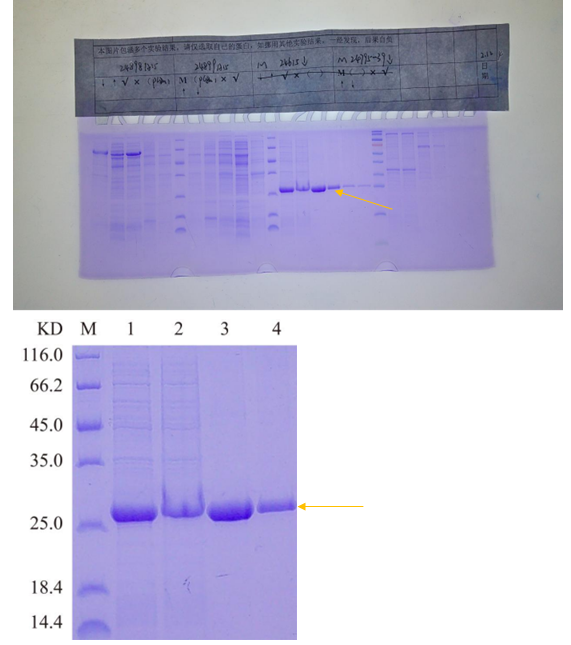


**Figure 6**. Original images of the expression and detection results of recombinant SAG17 and SAG23 proteins, among which the original image of recombinant protein SAG23 corresponds to Fig. 5C-3.


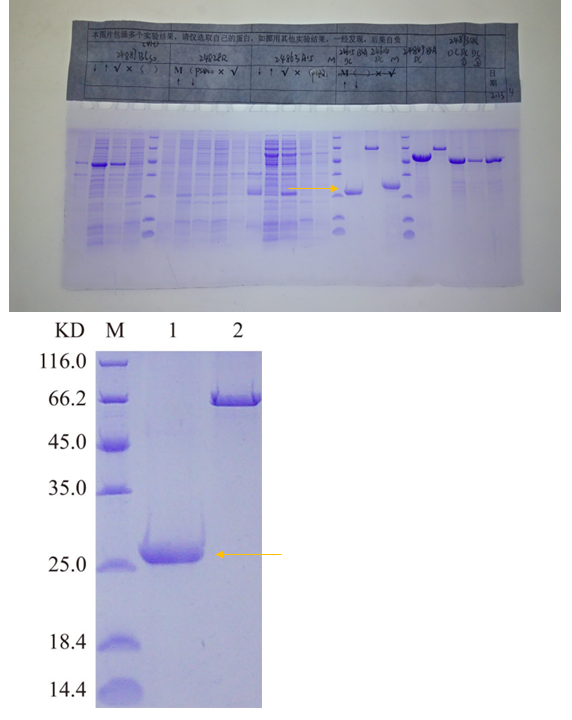


**Figure 7**. Original images of the expression and detection results of recombinant SAG17 and SAG23 proteins, among which the original image of recombinant protein SAG23 corresponds to Fig. 5C-4.


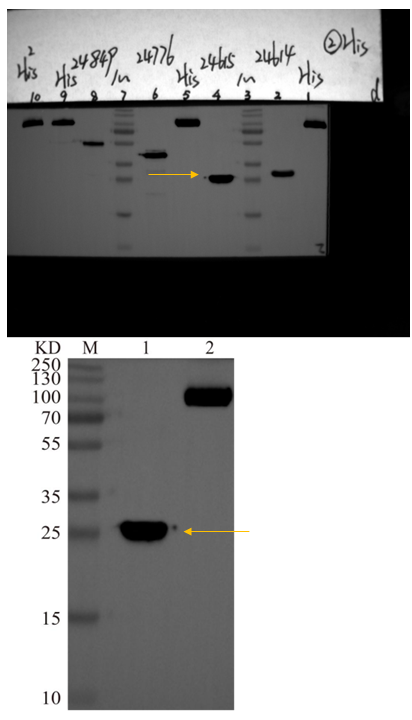


**Figure 8**. Original images of the expression and detection results of recombinant SAG17 and SAG23 proteins, among which the original image of recombinant protein SAG23 corresponds to Fig. 5C-5.
