# Supplementary material for: Integrative comparative genomics and transcriptomics reveal key roles of SAG17 and SAG23 in early-stage virulence divergence of Eimeria tenella
Source: Vet Res. 2026 Apr 28;57:86. doi: 10.1186/s13567-026-01730-0 (PMC13214288; doi:10.1186/s13567-026-01730-0)
Supplement: Supplementary file 4 — Additional file 4: Changes in OPG from 1 to 8 days post Eimeria tenella infection. [file 13567_2026_1730_MOESM4_ESM.docx]

Additional file 4. Changes in OPG from 1 to 8 days post *Eimeria tenella* infection

| Days post-infection (d) | GSIG-OPG (oocysts/g) | BSIG-OPG (oocysts/g) |
| --- | --- | --- |
| 1 | 0 | 0 |
| 2 | 0 | 0 |
| 3 | 0 | 0 |
| 4 | 0 | 1250 |
| 5 | 4250 | 15500 |
| 6 | 24750 | 45500 |
| 7 | 90960 | 150000 |
| 8 | 66000 | 98083 |

Note：Beijing strain infection group (BSIG). Guizhou strain infection group (GSIG). The results obtained during the preliminary oocyst propagation experiment.
